# Air-Stable, Large-Area 2D Metals and Semiconductors

**DOI:** 10.1021/acsnanoscienceau.3c00047

**Published:** 2024-01-30

**Authors:** Chengye Dong, Li-Syuan Lu, Yu-Chuan Lin, Joshua A. Robinson

**Affiliations:** † 2-Dimensional Crystal Consortium, The Pennsylvania State University, University Park, Pennsylvania 16802, United States; ‡ Department of Materials Science and Engineering, The Pennsylvania State University, University Park, Pennsylvania 16802, United States; § Department of Materials Science and Engineering, National Yang Ming Chiao Tung University, Hsinchu 300, Taiwan; ∥ Center for Atomically Thin Multifunctional Coatings, The Pennsylvania State University, University Park, Pennsylvania 16802, United States

**Keywords:** 2D Materials, 2D Metals, Transition Metal Dichalcogenides, Epitaxial Graphene, Confined Heteroepitaxy, Air Stability, Encapsulation, Surface Engineering

## Abstract

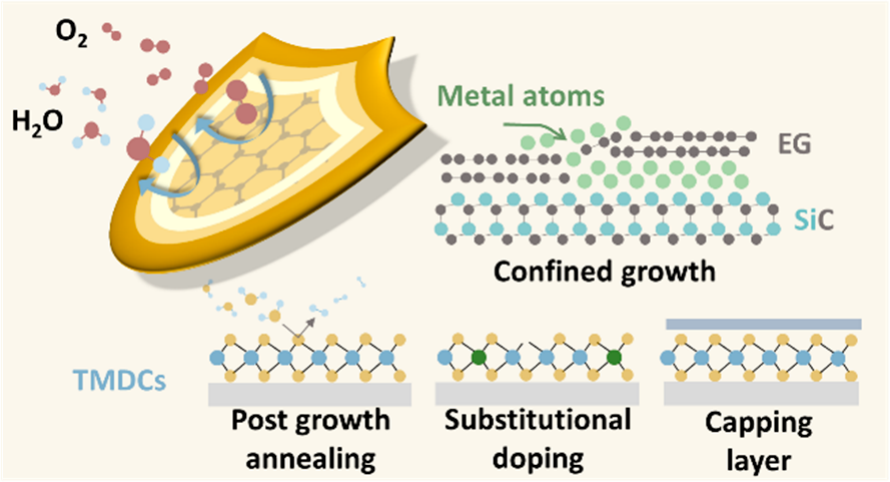

Two-dimensional (2D) materials are popular for fundamental physics study and technological applications in next-generation electronics, spintronics, and optoelectronic devices due to a wide range of intriguing physical and chemical properties. Recently, the family of 2D metals and 2D semiconductors has been expanding rapidly because they offer properties once unknown to us. One of the challenges to fully access their properties is poor stability in ambient conditions. In the first half of this Review, we briefly summarize common methods of preparing 2D metals and highlight some recent approaches for making air-stable 2D metals. Additionally, we introduce the physicochemical properties of some air-stable 2D metals recently explored. The second half discusses the air stability and oxidation mechanisms of 2D transition metal dichalcogenides and some elemental 2D semiconductors. Their air stability can be enhanced by optimizing growth temperature, substrates, and precursors during 2D material growth to improve material quality, which will be discussed. Other methods, including doping, postgrowth annealing, and encapsulation of insulators that can suppress defects and isolate the encapsulated samples from the ambient environment, will be reviewed.

## 1 Introduction

Two-dimensional (2D) materials have an essential place in nanoscience and technology because they have a wide range of compositions and properties across the full range wavelength from near-infrared to deep ultraviolet.^1,2^ The successful development of graphene and hexagonal boron nitride (hBN) monolayers encouraged extensive research on other 2D materials, such as transition metal dichalcogenides (TMDCs),^3^ and elemental 2D materials like phosphorene.^4^ One reason for the early success of graphene and hBN is their excellent thermal stability in ambient conditions,^5,6^ which eases the environmental conditions and enables rapid exploration of their properties and applications. While graphene (hBN) can be produced in a very large area with high quality, its semimetallic (insulating) band structure offers only a few applications in the visible wavelength range if they are not integrated with other semiconducting materials. Therefore, in the past decade, 2D materials research has continuously shifted to “beyond graphene (or hBN)” 2D materials^7^ including TMDC monolayers with electronic bandgaps.^8^

Recently, 2D metals have attracted tremendous research enthusiasm in exploring their novel fundamental physics phenomena and fascinating physiochemical properties. Some nonmagnetic elemental metals are predicted to exhibit magnetism in their 2D forms due to decreased coordination number and energy band narrowing in the out-of-plane orbitals.^9^ One-atom-thick Pb and In films are experimentally verified to be superconducting.^10^ Furthermore, their superconductivity in monolayer films originates from electron-photon interactions provided by both interlayer metallic and, more importantly, metal-substrate bonds. There are also several excellent review papers discussing the fabrication and application of 2D metals.^11−14^ All indicate that 2D metals have promising potential in quantum devices and spintronics, catalysis, sensing, energy storage, etc. However, compared to graphene, many beyond-graphene 2D materials are inferior in ambient stability because their constituents can react with water or oxygen and produce oxide compounds.^15^ Therefore, to fully utilize their potential, it is necessary to identify stable 2D materials and their stability via theory,^16^ understand the roles of defects and reactivity of 2D materials in surface oxidation,^17^ and establish strategies to preserve the quality of metastable 2D materials. For example, surface passivation/encapsulation is the most common measure against surface degradation for air-sensitive materials, as it has been demonstrated with graphene and hBN on metal surfaces.^6,18,19^ Removing surface defects and grain boundaries is also important since they likely will draw oxygen and carbon impurities to attach without being passivated.

Here, we will review the recent progress in making large-area air-stable atomically thin metals and TMDCs by bottom-up and top-down techniques. First, we provide a brief overview of 2D metal research and introduce confined heteroepitaxy for the intercalation of a series of 2D transition and group-III to -V metal elements at the epitaxial graphene/SiC interface that isolates the atomically thin metals from the ambient environment.^20,21^ Due to graphene’s transparency, we can characterize the intrinsic properties of “half-van der Waals” 2D metals through the topmost encapsulating epitaxial graphene. Next, the recent development of scalable, high-quality 2D TMDC semiconductors grown by chemical vapor deposition (CVD) will be reviewed. First, we briefly review the stability of 2D TMDCs with different chalcogen elements (O, S, Se, and Te). Second, we show that, by controlling the parameters of CVD and choosing appropriate precursors and templates, grain boundary and intrinsic defects of deposited TMDC films can be suppressed and, in turn, their ability against oxidation and impurity incorporation can be improved. At the end of this review, we will discuss their future opportunities and possible applications of integrating both materials.

## 2 Discussion

### 2.1 Making Air-Stable 2D Metals

Due to the nondirectional metallic bonding, monoelemental metals or alloys always prefer to form three-dimensional (3D) structures, resulting in a big challenge to synthesize their two-dimensional (2D) counterparts.^22,23^ To date, a number of preparation methods have been developed to fabricate ultrathin 2D metals, such as solution-based chemical methods, including hydrothermal/solvothermal synthesis,^24−28^ surfactant/ligand assisted synthesis,^29−34^ seeded growth,^35−38^ template-confined growth,^39−45^ and others,^46,47^ molecular beam evaporation (MBE),^48−53^ mechanical compression/rolling,^54−56^ polymer surface buckling enabled exfoliation (PSBEE),^57^ and liquid exfoliation.^58−60^ For instance, solution-based chemical methods, as the most popular methods to synthesize 2D metals for sensing, catalysis, and other applications due to their high yield and low cost, can offer reliable approaches via complex chemical procedures to prepare metal films with mono/multiple atomic thickness.^11,61^ However, the lateral size of 2D metals is always less than micrometers which limits their applications. Additionally, the surfactants or solution used in the procedure may functionalize these metal films, resulting in the degradation of their properties. Notably, all the aforementioned methods have their own merits but share the same shortcoming that 2D metals are sensitive to the ambient atmosphere and can be easily oxidized.^62^ Although postdeposition or coating of the protective layer can isolate metal films from air or chemical environments, the additional step will bring up more issues in terms of accessibility of 2D metals, contamination, and cost. Owing to their impermeability to liquid and gas, chemical and thermal stability, graphene and its derivatives are widely explored as corrosion protecting coatings on metals.^63^ Moreover, graphene is well-known as an optically transparent film for optical devices.^64,65^ Recent works also verified that graphene could act as a transparent layer to the potential field of many substrates, which enables remote epitaxial growth of III–V semiconductors,^66,67^ halide perovskites,^68^ and others.^69,70^

By heating silicon carbide (SiC) substrate to a temperature higher than 1200 °C under pressure ranging from ultrahigh vacuum to atmospheric pressure, silicon (Si) atoms will sublimate from the (0001) face of the substrate and remaining carbon atoms will go through a  reconstruction, resulting in the formation of a carbon buffer layer and one graphene layer.^71−73^ The buffer layer is underneath graphene and partially bonded to SiC. When epitaxial graphene (EG) on SiC is annealed in H_2_, H atoms will penetrate through defects and boundaries in EG and intercalate into the space between the buffer layer and SiC, decoupling the buffer layer from SiC and forming an additional graphene layer.^74,75^ In this way, the intercalants will be sealed in the interface and isolated from ambient by capping graphene. Inspired by the hydrogen intercalation at EG/SiC interface, many efforts have been made to intercalate metal atoms into the EG/SiC interface to form confined 2D metals. Most of the confined growths of 2D metals are achieved by intercalation under ultrahigh vacuum (UHV).^76−117^ First, metal atoms are deposited on the buffer or EG surface via thermal or E-beam evaporation, sputtering, or MBE. Then metal/EG/SiC is annealed under vacuum and metal atoms will enter EG/SiC interface. In this process, intrinsic defects or boundaries in buffer and EG play an important role in trapping the metal atoms on the surface, driving them into the interface, detaching from defects and diffusing in the interface to form 2D metals during thermal treatment of metal/EG/SiC.^76−78^ Taking advantage of in situ characterization methods, such as scanning tunneling microscopy (STM), low-energy electron diffraction (LEED), and angle-resolved photoemission spectroscopy (ARPES), the structures of 2D metals and band structure evolution of EG/2D metal heterostructures have been extensively explored. For example, Rosenzweig et al. revealed that intercalated Yb at 250 °C forms a disordered phase and transitions to a  configuration with respect to the graphene layer when intercalating at 450 °C.^76^ Moreover, Yb intercalation can induce an extreme n-type doping level of 3.5 × 10^14^ cm^–2^ in EG, which is high enough to reach van Hove singularity. However, 2D metals prepared by confined growth under UHV are always patches with lateral scale less than micrometers due to the limitations of defect density.

**Figure 1:**
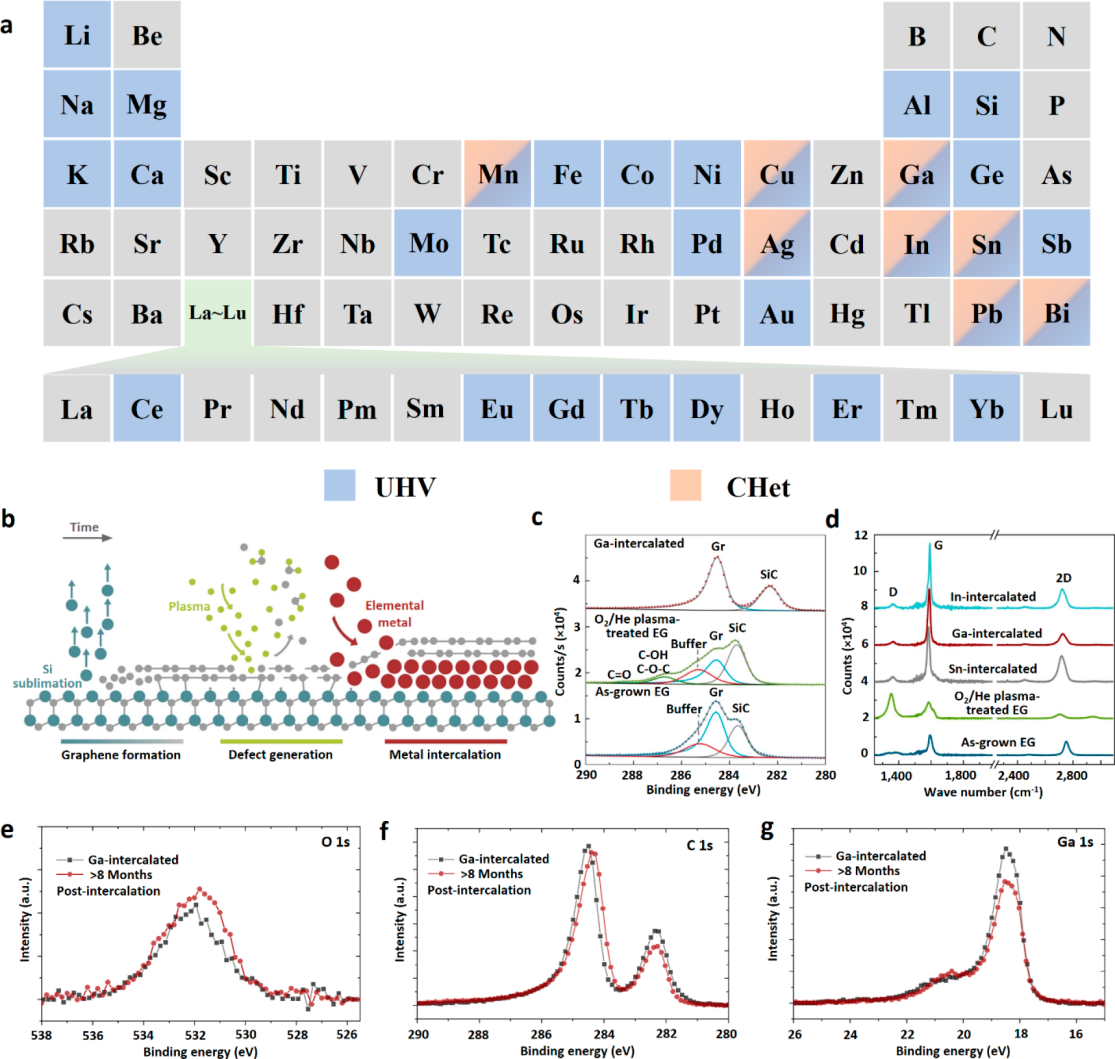
(a) Summary of 2D metals prepared via confined growth between SiC and graphene. (b) Schematics of CHet process. (c, d) XPS and Raman evolution during CHet process, respectively. The defects generated from plasma etching are healed after metal intercalation in the interface, which will prevent oxidation of metals. (e, f) XPS spectra of Ga intercalated EG postsynthesis (within several days) and >8 months later, indicating the excellent air-stability of 2D Ga synthesized via CHet.^77^ (b–g) Adapted with permission from ref (77). Copyright 2020 Springer Nature.

To synthesize large-scale, single-crystal, and environmentally stable atomically thin metals, a growth strategy, dubbed “Confinement Heteroepitaxy (CHet)”, was developed to synthesize 2D metals.^77^ In CHet (shown in Figure 1b), atomic vacancies are introduced in epitaxial graphene by O_2_ plasma treatment. Elemental metals are subsequently intercalated into the EG/SiC interface through these vacancies. Finally, these defects will be healed during the process, and graphene becomes a confining layer for the intercalated 2D metals. XPS and Raman spectroscopy data (Figure 1c, d) can be used to describe the process. XPS spectra indicate the formation of C=O, C–OH, and C–O–C bonds in the C 1s spectrum after O_2_ plasma treatment. In addition, the D band of graphene Raman spectra arises after the plasma treatment due to defect formation. After metals are intercalated, the oxygen functional groups attached to graphene are removed and the graphene D bands are suppressed, indicating the quality of graphene is recovered by the end of the CHet process. Like H intercalation, decoupling of Si–C and C–C bonds in the C 1s spectrum also indicates the buffer layer is released by metal intercalation. After examining the XPS spectrum of a Ga intercalated EG sample placed in ambient for 8 months, 2D Ga prepared via CHet shows minimal change, which reveals the excellent air-stability of CHet-synthesized 2D metals. The summary of recent progress in fabrication of 2D metals via CHet and confined growth under UHV is shown in Figure 1a. Furthermore, the properties of these air-stable 2D metals prepared via confined growth will be discussed in the following sections.

### 2.2 Properties of Air-Stable 2D Metals

#### 2.2.1 Electronic Properties

**Figure 2:**
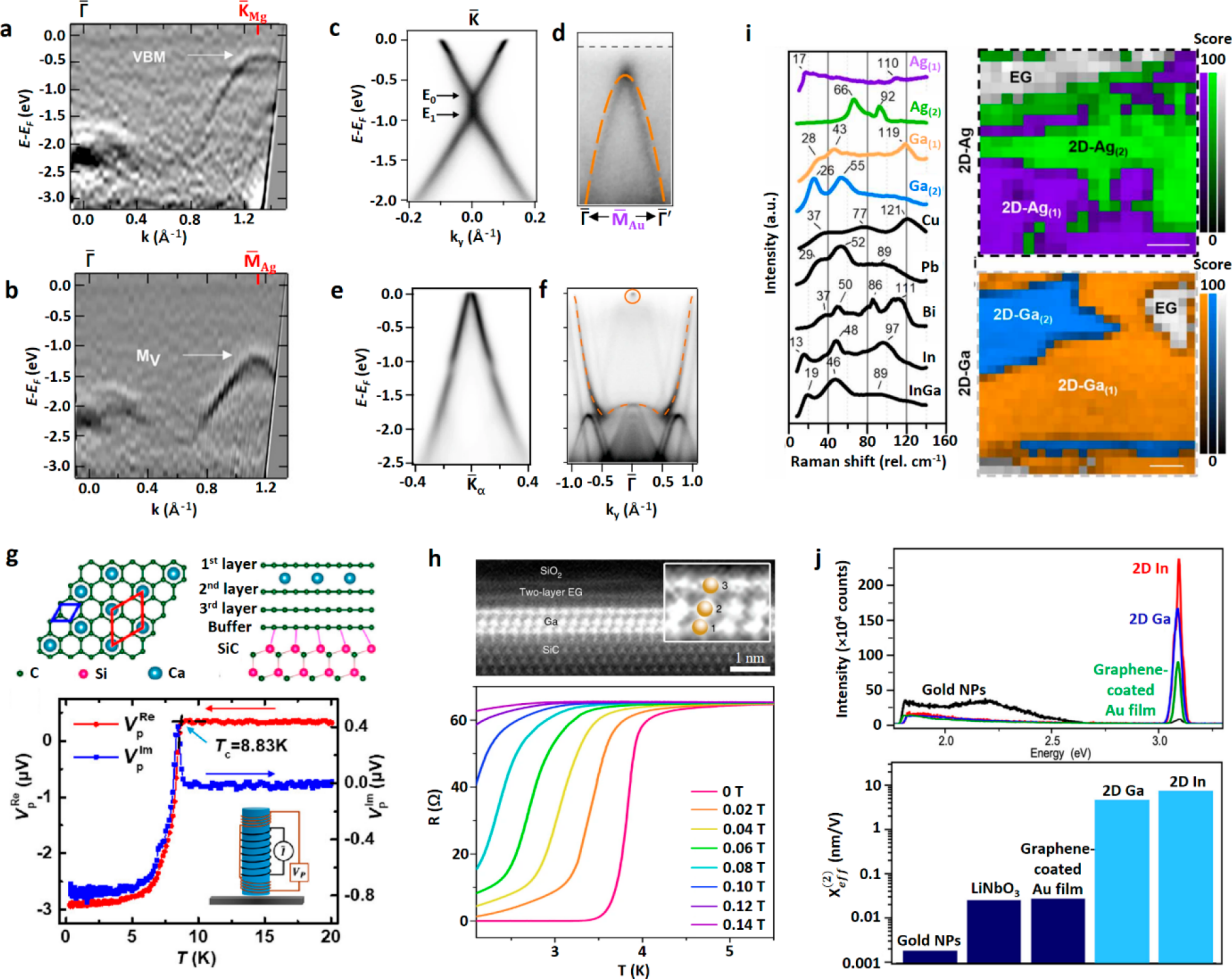
(a ,b) Second derivative images of occupied and unoccupied electronic structures of monolayer Ag, respectively.^79^ Adapted with permission from ref (79). Copyright 2022 American Chemical Society. (c, d) Graphene and Au band dispersion after monolayer Au intercalated in EG/SiC, respectively.^80^ (e,f) Graphene and Au band dispersion after bilayer Au intercalated in EG/SiC, respectively.^80^ (c–f) Adapted with permission under a Creative Commons CC-BY License from ref (80). Copyright 2020 Springer Nature. (g, h) Superconducting properties of 2D Ca and Ga, respectively.^77,85^ Adapted with permission from refs (77) and (85). Copyright 2020 Springer Nature and 2022 American Chemical Society, respectively. (i) Ultralow frequency fingerprint of 2D metals and Ga/Ag with different phases.^87^ Adapted with permission from ref (87). Copyright 2021 IOP publishing. (j) Nonlinear spectra and of χ^(2)^ values for 2D metals and 3D gold nanoparticles.^90^ Adapted with permission from ref (90). Copyright 2020 American Chemical Society.

Compared to their 3D counterpart, the confined 2D metal layers between EG/SiC interface display intriguingly electronic properties due to their half-vdW structure, in which the bonding character transits with high internal gradient from covalent bonding between bottom metal layer and SiC, to interlayer metallic bonding and to vdW bonding between top metal layer and EG. Exemplarily, first-principles calculations have predicted that monolayer silver (Ag) and gold (Au) are semiconductors owing to hybridization with the SiC surface.^78^ Recently, Lee et al. experimentally measured the band gap of monolayer Ag confined between bilayer graphene and SiC after the CHet process.^79^ According to static-ARPES data on occupied band dispersions of monolayer Ag (Figure 2a, b), the valence band maximum (VBM) is identified at *K̅*_Ag_ with a binding energy of ∼0.45 eV below the Fermi level (*E*_F_). Then the unoccupied band dispersions near Γ̅ revealed by time-resolved ARPES shows the conduction band minimum (CBM) is identified at ∼0.56 eV above *E*_F_. Therefore, the monolayer Ag is verified to be a semiconductor with an indirect band gap of ∼1 eV, which is significantly higher than the earlier density functional theory (DFT) prediction of 0.2 eV. Forti et al. also experimentally verified that monolayer Au confined at the EG/SiC interface is a semiconductor for which the VBM is located 50 meV below *E*_F_ (Figure 2c–f).^80^ The monolayer Au acts as the electron donor to graphene and drives the Dirac point *E*_0_ to 700 meV under *E*_F_. Notably, 2D semiconducting Au would become metallic when the thickness of Au increases by one atomic layer, lifting the Dirac cone of graphene 150 meV above *E*_F_.

#### 2.2.2 Superconductivity

While pristine EG on SiC and bulk metals exhibit poor superconductivity, some 2D metals confined in EG/SiC exhibit enhanced superconductivity. Ca intercalated EG is one of these systems that has stimulated considerable research interest. The superconductivity of Ca intercalated EG originates from the formation of C_6_CaC_6_ between freestanding graphene layers.^81−85^ Wang et al. revealed that the emerging superconductivity in C_6_CaC_6_ is mainly due to the free-electron-like interlayer band merging into π* bands around *E*_F_.^85^ Notably, Figure 2g shows that the critical temperature at 8.83 K is obtained via intercalating Ca into the top two graphene layers in a trilayer EG system and can be attributed to the suppression of charge-density wave by screening C_6_CaC_6_ film from the buffer layer with a graphene layer. Briggs et al. developed CHet to successfully obtain 2–3 layers of Ga, which is covalently bonded to the bottom SiC and has vdW mediated bonds with the graphene overlayer.^77^ The novel “half-vdW” Ga is verified to have a 4 times higher critical temperature close to 4 K than its bulk counterpart, as shown in Figure 2h.

#### 2.2.3 Optical Properties

Raman spectroscopy is one of the most important characterization techniques, which can supply abundant information on 2D materials including layer numbers, interlayer coupling and layer stacking configuration.^86^ Wetherington et al.^87^ explored the unique low-frequency (LF, < 100 cm^–1^) Raman features of 2D polar metals (Ag, Cu, Pb, Bi, Ga, In) and alloys (In_*x*_Ga_1–*x*_) synthesized via CHet, displayed in Figure 2i.^123^ It is revealed that these LF Raman features are related to metal atoms stabilized in the interface and also the phase of 2D metals. Taking 2D Ga as an example and combining with DFT studies, the origination of these peaks contributes to the surface resonance mode coupled to SiC phonons in EG/2D metal/SiC system.^88,89^ The covalent bond between the bottom 2D metal layer and SiC also plays a significant role in the resonant Raman enhancement of the shear modes. Besides unique Raman features, 2D polar metals confined in EG/SiC interface display remarkable nonlinear optical properties.^90−92^ Steves et al. reported that 2D Ga and In are two of the most efficient second harmonic generation systems, with room-temperature near-infrared χ^(2)^ values approaching 10 nm/V.^90^ The surprisingly large χ^(2)^ value, which is over 100× larger than 3D gold nanorods, graphene-coated Au films, and LiNbO_3_, is due to symmetry breaking in atomically thin 2D metal films as a result of the bonding environment transition from the covalent bonds at the SiC/2D metal interface to the vdW ones at the 2D metal/graphene interface (Figure 2j).

### 2.3 Improving Air-Stability of 2D Semiconductors

#### 2.3.1 Previous Investigations

**Figure 3:**
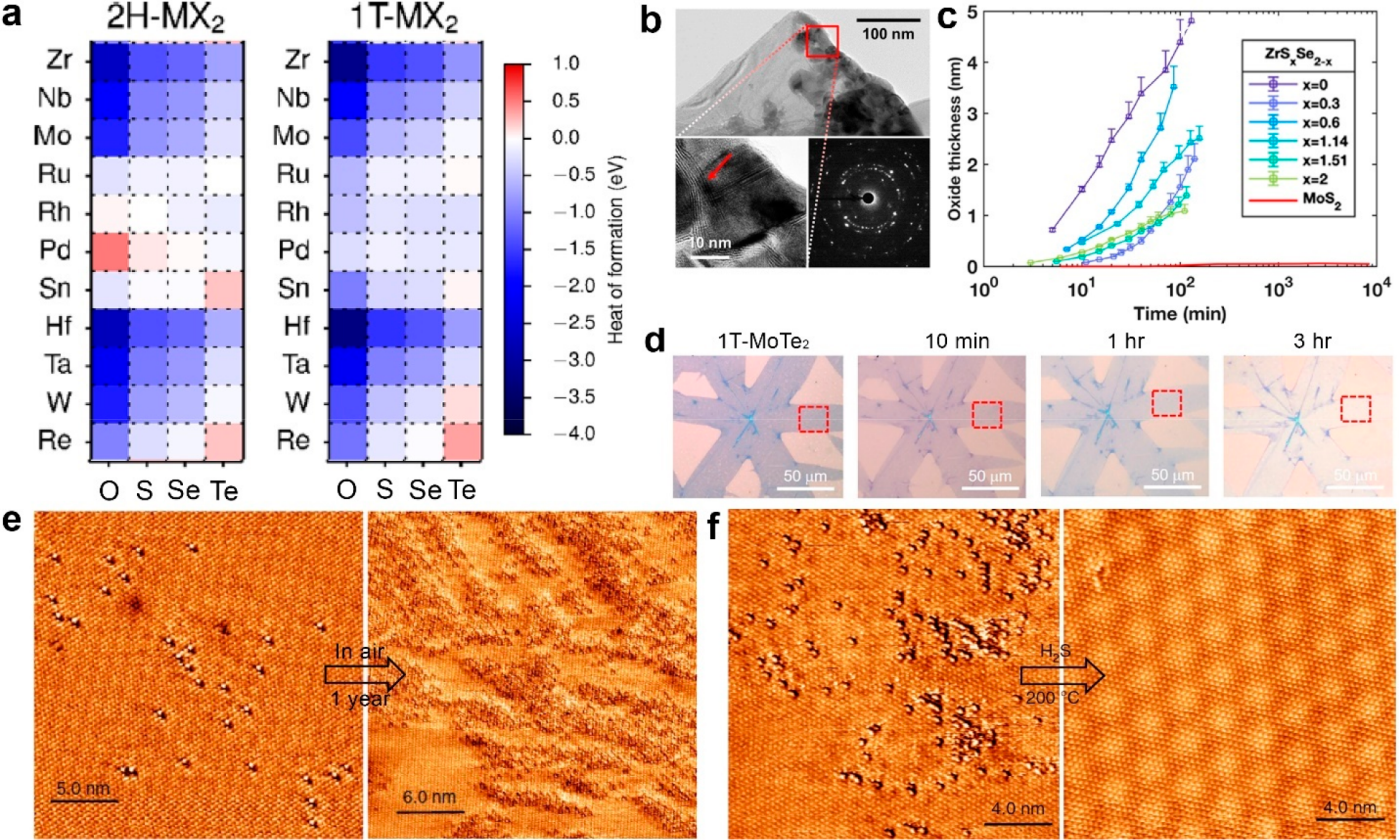
(a) Calculated heat of formation for monolayers in the 2H and 1T phases with selected transition metals. In all chalcogen elements, oxide provides the highest stability.^16^ Adapted with permission from ref (16). Copyright 2015 American Chemical Society. (b) TEM and SAED images of an oxidized few-layer HfS_2_. The oxidized HfS_2_ appears to be polycrystalline. Adapted with permission from ref (96). Copyright 2016 American Chemical Society. (c) Kinetics of native oxide formation on freshly cleaved ZrS_*x*_Se_2–x_ with x from 0 to 0.3, 0.6, 1.14, 1.51, 2, and MoS_2_ crystals were studied with spectroscopic ellipsometry measurements. Oxide thickness versus exposure time plots for ZrS_*x*_Se_2–x_ and MoS_2_ crystals show that Zr–S and Zr–Se switch to Zr–O bonds rapidly under ambient conditions. Adapted with permission from ref (98). Copyright 2020 American Chemical Society. (d) Optical images of 1T-MoTe_2_ exposed in air after its growth and after 10 min, 1 h, and 3 h. A clear dimming of the contrast inside the dashed red box is visible and corresponds to about 92% contrast intensity loss in 3 h.^99^ Adapted with permission from ref (99). Copyright 2021 American Chemical Society. (e) Atomic-resolution STM images (5 mV, 2 nA) of an exfoliated MoS_2_ monolayer on Au after 1 month (left) and 1 year (right) of ambient exposure, revealing a progressive defect formation.^101^ (f) Reduction of 2D oxidized MoS_2_ to pristine MoS_2_: Representative atomic-resolution STM images (5 mV, 2 nA) of 2D MoS_2–x_O_*x*_ before (left) and after (right) 30 min annealing with H_2_S at 200 °C, showing the reduction of the oxy-sulfide solid solution to the pristine MoS_2_ through resubstitution of O by S.^101^ (e, f) Adapted with permission from ref (101). Copyright 2018 Springer Nature.

Two-dimensional (2D) semiconductors are promising candidates for next-generation transistors and diodes because they can overcome the fundamental material and device challenges that Si is facing when the channel body needs to be thinner than 5 nm.^93^ To adopt them in practical optoelectronic applications in the foreseeable future, 2D semiconductors that can be synthesized and stable enough in ambient conditions should be first to be considered. There is a large variety of 2D semiconductors with bandgaps ranging from the infrared to the ultraviolet, including TMDCs,^3^ elemental monolayers (phosphorene and silicene),^4^ and group III–VI metal chalcogenides (GaSe and InSe).^94^ Computational study is helpful for understanding the ambient stability of some of 2D semiconductors.^16,95^ Rasmussen et al.^16^ tested 2H- and 1T-phase TMDCs with a variety of combinations of transition metal and chalcogen atoms using density functional theory calculations (Figure 3a). Taking Hf- and Zr-based TMDCs as an example, their formation energies indicate that their oxide form is the most stable. Compared to other transition metals, their formation energy with oxide is more negative than that of other transition metals, such as W and Mo. In 2016, Chael et al. already observed oxidation on HfS_2_ crystals under ambient conditions within a few hours and found that oxidation starts from edge part of HfS_2_ in TEM experiments (Figure 3b).^96^ The work by Mleczko et al.^97^ found that exfoliated ZrSe_2_ and HfSe_2_ crystals oxidize rapidly even in a diluted O_2_ environment to form ZrO_*x*_/ZrSe_2_ and HfO_*x*_/HfSe_2_. The rapid oxidation of ZrS_*x*_Se_2-x_ alloys was investigated with real-time spectroscopic ellipsometry measurements (Figure 3c) and molecular dynamic simulations by Jo et al. and was attributed to favorable O_2_ adsorption on ZrS_*x*_Se_2–*x*_ and Zr–O bond switching that reduce the structural stability.^98^ They also found that oxidation of ZrS_*x*_Se_2-x_ exacerbates with increasing Se fraction. Taking MoTe_2_ in H- and T-phase as another example in Figure 3a, they are less stable compared to their MoO_2_ counterparts and can be oxidized faster than MoS_2_ and MoSe_2_ in ambient conditions. Pace et al.^99^ synthesized 1T-MoTe_2_ on SiO_2_/Si and used optical microscopy (OM) image contrast to observe real-time oxidation of MoTe_2_ flakes in air (Figure 3d). They saw noticeable optical contrast change and increased roughness in air within 10 min and severe material degradation in 3 h. The above examples show that layered materials may be driven to oxidation by their thermodynamic instability even when they are made with the highest quality.^137^

Rasmussen et al.^16^ also reported that the formation energies of group-VI TMDC including MoS_2_, MoSe_2_, WS_2_, and WSe_2_ are not largely different from their oxide form, indicating their oxidation may not occur as rapidly as Zr- and Hf-based TMDC. Their excellent stability was experimentally confirmed by their device performance in ambient conditions in numerous works.^100^ Jo et al. demonstrated the evidence of the air stability via spectral ellipsometry, no spontaneous native oxidized layer was observed over 6 days, and they conducted XPS measurement on MoS_2_ cleaved for over a year and found that no elevated oxygen levels were observed.^98^ However, a surface science study performed on group-VI TMDC indicates that they are not always free from oxidation. Pető et al.^101^ studied surface oxidation of MoS_2_ and MoSe_2_ in a year with scanning tunneling microscopy found that oxygen gradually reacts with MoS_2_ surface to form volatile gaseous SO_2_ and heavily oxidize MoS_2_ into MoO_*x*_ over time (Figure 3e) while MoSe_2_ remains mostly intact due to a high energy barrier for SeO_2_ formation. In addition, oxidation and impurity substitution can occur at chalcogen vacancies and grain boundaries where chemical reactivity is high.^102^ Pető et al.^101^ also demonstrated that the surface oxidation and point defects of 2D MoS_2_ can be healed by conducting thermal annealing with H_2_S at 200 °C (Figure 3f), which can be integrated into the thin film processes of these 2D semiconductors. Despite the DFT calculation guideline revealing that the stability of sulfur-based TMDC is better than selenium-based TMDC, the formation energy of S–O and Se–O bonds also needs to be carefully considered when exposed in an ambient condition.

#### 2.3.2 Bottom-Up Approaches to Improve the Air Stability of 2D Semiconductors

Among all 2D semiconductors, W- and Mo-based 2D TMDCs have been at the center of the research and development for the implementation of practical 2D semiconductors because they have intriguing properties including high ON/OFF ratios, excellent flexibility, and direct bandgaps and can grow on various substrates. Although there are other stable TMDC semiconductors like PdSe_2_^103^ and PtSe_2_,^104^ large-area controllable growth and characterization of group-VI TMDC films are so far the most advanced and mature. In the past few years, metalorganic chemical vapor deposition (MOCVD) has demonstrated its capabilities to synthesize large-area electronic-grade 2D semiconductors.^105−107^ Growth substrate plays an important role in making good and air-stable films. C-plane sapphire is an ideal template for MoS_2_ and WSe_2_ growth because it can help TMDC grow into a film with long-range order and minimize grain boundaries through vdW epitaxy.^107^ Precursor consideration is not trivial. For example, Eichfeld et al.^106^ reported a MOCVD process for WSe_2_ crystals, in which W(CO)_6_ and (CH_3_)_2_Se were chosen as the precursors to provide W and Se. However, the purity of the precursors and carbon from C_2_H_6_Se were found to impact the film quality drastically by promoting defect densities, grain boundaries, and impurities that will eventually oxidize. Therefore, for the next generation of MOCVD for WSe_2_, the Se supply was switched from (CH_3_)_2_Se to H_2_Se gas that was proved to be cleaner and release less carbon during the process.^107^

**Figure 4:**
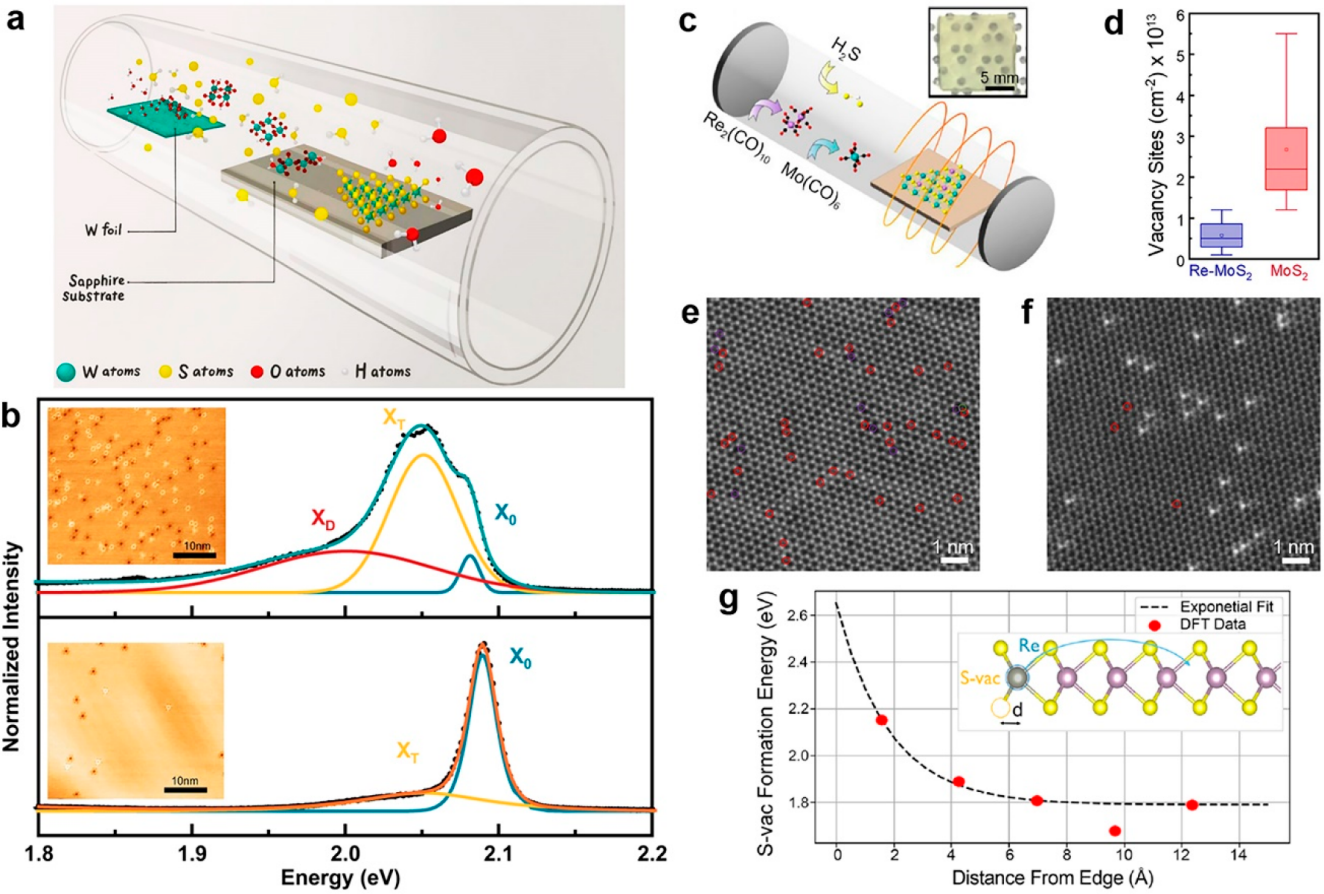
(a) Schematic of hydroxide vapor phase deposition (OHVPD) growth of WS_2_ monolayers.^108^ (b) Low-temperature PL spectra and STM topography (inset) of CVD- (top) OHVPD- (bottom) WS_2_ monolayers provide the comparison of their surface defect density and defect-bound exciton. The solid lines and dashed ones are the experimental and fitted peaks, respectively. The fitted peaks can be assigned to neutral exciton (X_0_), trion (X_T_), and defect-bound exciton (X_D_).^108^ Adapted with permission under a Creative Commons CC-BY License from ref (108). Copyright 2022 Springer Nature. (c) Illustration of the growth of ML Re-MoS_2_ using metalorganic precursors. Inset: a camera image of a Re-MoS_2_ film on sapphire. (d) Statistic for sulfur vacancy sites in MoS_2_ and Re-MoS_2_ based on TEM study. (e, f) Point defect density. (e) MoS_2_, and (f) 5 atom % Re-MoS_2_ indicates their S-site defect (marked with red circles) densities using Z-contrast STEM image. In (e), there are 34 single-sulfur vacancies and 11 double-sulfur vacancies while in (f), there are 3 single-sulfur vacancies. The brighter atoms in (f) are Re due to its larger Z number. (g) DFT model of sulfur vacancy formation energy as a function of the distance between the sulfur vacancy at the edge of MoS_2_ model and the Re position moving away from the edge. The corresponding energy values as a function of the Re position are listed. (c–g) Adapted with permission from ref (110). Copyright 2023 American Chemical Society.

One reason that the electronic industry may prefer MOCVD over powder-based CVD is its scalability. For small-scale lab-based research, powder-based CVD (PCVD) is still useful because it can provide prototypical experiments in a short time and is budget-friendly. The common precursors for PCVD of group-VI TMDC include MoO_3_, WO_3_, S, Se, and Te powders. However, TMDC flakes grown with oxide precursors typically have abundant defects and oxidation and ultimately exhibit insufficient performance for advanced devices. A recent result shows that using H_2_O as the transport agent during synthesis of WS_2_ can provide positive outcomes. Wan et al.^108^ developed hydroxide vapor phase deposition (OHVPD) that includes H_2_O in the growth of 2D WS_2_ on sapphire (Figure 4a). The authors found that W–OH bond in the hydroxide intermediates produced from the reaction of H_2_O and a high-purity W foil makes WS_2_ formation more energetically favorable than W–O bond from the use of WO_3_. Unlike the direct reaction of WO_3_ and S vapors, the OHVPD relies on H_2_O vapors to slowly oxidize W foil and carry high-purity W clusters to reduce excessive oxygen and other impurities in the deposited WS_2_ crystals. To compare the quality between traditional CVD- and OHVPD-grown WS_2_, STM and low temperature (4 K) photoluminescence (PL) experiments were performed on both samples (Figure 4b). The defect count carried out in their STM images (inset, Figure 4b) shows the total atomic defect number was reduced from near 21 000 in CVD-grown WS_2_ to 8000 in the OHVPD-grown one. Furthermore, low-temperature PL spectra corroborate with the STM data, showing that the emission of defect-bound exciton (X_D_) of OHVPD-grown WS_2_ at 2 eV is suppressed by at least 4 folds and less emission of trions, compared to CVD-grown WS_2_.

Doping and alloying TMDC could help to reduce the chalcogen point defects of TMDC. Li et al.^109^ conducted powder-based CVD using mixture of WO_3_ and MoO_3_ with predetermined ratios to synthesize W_1–*x*_Mo_*x*_Se_2_ and found that adding W into MoSe_2_ can suppress the original Se vacancies by nearly half. STEM analysis performed on 5000 Se sites in the STEM images of MoSe_2_ and W_0.18_Mo_0.82_Se_2_ shows that the percentage of Se vacancies was reduced from (4 ± 0.06)% to (2 ± 0.08)%.^109^ This phenomenon was attributed to a stronger bonding strength and higher formation energy of Se vacancy in the presence of W in MoSe_2_.^110^ However, it was not rigorously verified. Low-temperature PL measurements were carried out on MoSe_2_ and W_0.18_Mo_0.82_Se_2_ to understand the impact of the W dopant on the optical properties. While doping W does not change the emission of free excitons and trions (band 1,2 at 1.6 and 1.58 eV, respectively), it reduces the emission of X_D_ at around 1.5 eV by 4-fold.^109^ A probable mechanism for defect reduction in TMDCs enabled by dopant incorporation was recently examined in another work using Re-doped MoS_2_ (Re-MoS_2_). Torsi et al.^110^ synthesized Re-MoS_2_ via MOCVD using Re_2_(CO)_10_ and Mo(CO)_6_ on sapphire (Figure 4c). They produced Re substitutional doping ranging from 0.1 to 5 atomic percent (atom %) and found that the sulfur vacancy (S_v_) can be reduced when Re is present during growth. The statistical study shows that the density can be reduced from 3 × 10^13^ cm^–2^ in MoS_2_ to 5 × 10^12^ cm^–2^ in Re-MoS_2_. The TEM experiments found that the sample had a high density of single- and double-S vacancies in undoped MoS_2_ (Figure 4e), while there’s only few single sulfur vacancies were found when Re was introduced into the sample (Figure 4f). To explain the defect reduction that occurs during Re-MoS_2_ growth, energetic models to study the influence of present Re in MoS_2_ lattice to sulfur vacancy formation were constructed by DFT calculations (Figure 4g). The theory shows that the formation energy for single-sulfur vacancy is the largest when the Re is present at the nearest cation site and can apply to the actual growth in which Re attached to the growing edges of MoS_2_ domains can make sulfur vacancy harder to incorporate into the edges.

#### 2.3.3 Defect Healing and Passivation for Air Stability Improvement

**Figure 5:**
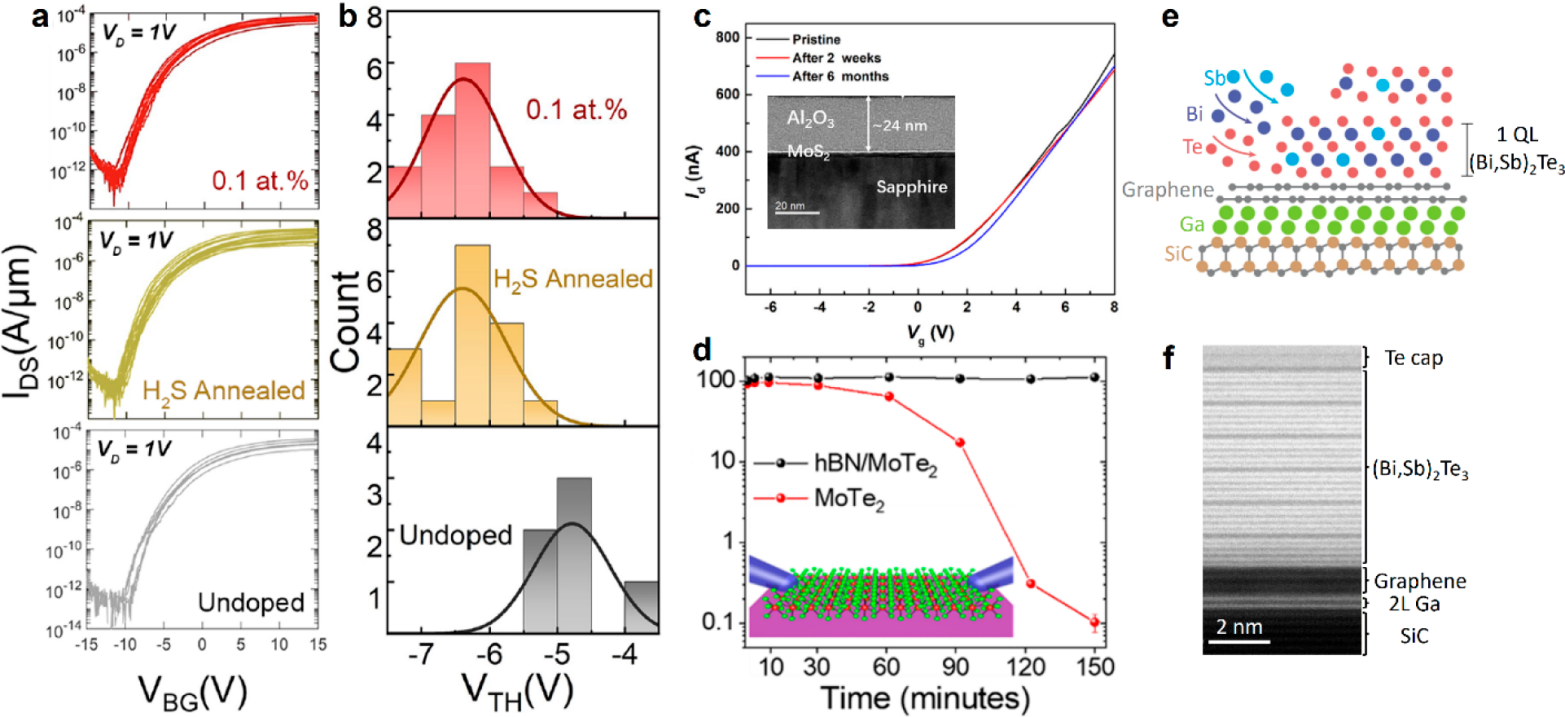
(a) Transfer characteristics for undoped, H_2_S annealed, and 0.1 atom % Re-MoS_2_ FETs measured in ambient conditions and their (b) statistical analysis of *V*_TH_ at *V*_DS_ = 1 V The red horizontal line in each graph marks *I*_DS_ at 10^–5^ A/mm. (a, b) Adapted and modified with permission from ref (110). Copyright 2023 American Chemical Society. (c) Time-dependent study (pristine, 2 weeks, and 6 months) of transfer characteristics of a top-gated MoS_2_ FET using 24 nm Al_2_O_3_ as the encapsulation and dielectric. Inset shows the cross-sectional view of the device. Adapted with permission from ref (115). Copyright 2022 Elsevier. (d) Variation of the current flowing over 150 min through a single MoTe_2_ crystal exposed in air (red dots) and encapsulated with hBN (black dots) at *V*_DS_ = 0.1 V. Adapted and modified with permission from ref (99). Copyright 2021 American Chemical Society. (e, f) Structure of (Bi,Sb)_2_Te_3_/graphene/gallium (BST/Gr/Ga) thin films. Cross-sectional TEM shows the clean interface in the heterostructures.^116^ (e, f) Adapted with permission from ref (116). Copyright 2023 Springer Nature.

Defects exacerbate oxidation of 2D materials in air and ultimately impact the electrical properties and lifetime of the devices including field-effect transistor (FET). Molybdenum oxides (MoO_*x*_) are p-type dopants to MoS_2_^111^ and a p-type material^112^ and can form when oxygen fills into sulfur vacancies of MoS_2_.^113^Figure 5a shows transfer characteristics of 3 types of MoS_2_ FET that depend on back-gate voltage (*V*_BG_): undoped, annealed with H_2_S, and 0.1 atom % Re-doped MoS_2_ under a drain voltage (*V*_d_) of 1 V. The undoped MoS_2_ FET has a smaller on-current density because its defect density is the highest without intentional defect suppression. H_2_S-annealed MoS_2_ FET was made of undoped MoS_2_ that was postgrowth annealed at 500 °C with H_2_S to heal sulfur vacancy with excessive sulfur. Compared to undoped MoS_2_ FET, it has a slightly better on-current density and more negative threshold voltage (*V*_th_) (Figure 5b); both should be considered as evidence for sulfur vacancy removal.^110,113^ 0.1 atom % Re-MoS_2_ FET also exhibits a V_th_ distribution similar to the H_2_S-annealed FET and the highest on-current density, indicating that Re not only helps suppress sulfur vacancy (similar to the effect achieved by H_2_S postgrowth annealing) but also improves the contact resistance by electrical doping. Encapsulation is useful to isolate 2D materials from the ambient and can be conveniently integrated into device fabrication processes. To prevent the direct contact of metastable 2D materials such as ZrSe_2_, HfSe_2_, MoTe_2_ and black phosphorus (BP) with the ambient, surface encapsulation with hBN or oxides can be deposited on the surfaces by atomic layer deposition (ALD) at low temperature (≤200 °C). An experiment by Kim et al.^114^ observed the change in the sheet resistance (*R*_SH_) of two BP flakes with and without Al_2_O_3_ encapsulation in air over 1 week. Their result indicates the *R*_SH_ of unencapsulated BP increases as a result of oxidation that occurs from the edge of the flake and propagates into the center of the flake. On the other hand, the BP whose edges and surface are encapsulated shows that its *R*_SH_ remains constant over a week. In a recent study by Zhao et al.,^115^ a MoS_2_ FET capped with 24 nm Al_2_O_3_ prepared by ALD exhibits unchanged transport characteristics over 6 months (Figure 5c). This encapsulation can prevent 2D material surface oxidation and block water and oxygen intercalating into the device interfaces of 2D materials/dielectric^115^ and/2D materials/substrate. There is a valid concern about using water in ALD of oxides (Al_2_O_3_ and HfO_2_) for encapsulation since there is a chance that water may react with surface defects of 2D materials even at room temperature. The alternative to oxide is hBN that can be prepared on 2D surfaces by transfer. Pace et al.^99^ transferred CVD-grown hBN onto 1T-MoTe_2_ devices to demonstrate the benefit of encapsulation (Figure 5d). The current monitored by two-terminal electrical measurement dropped 1000 times within 150 min without hBN encapsulation, while the current with encapsulation remained constant. BN can also be put down by pulsed lased positioning or CVD so that any contact with solvent can be avoided.

## 3 Summary and Future Work

In this Review, we discuss the instability of large-area 2D metals and 2D semiconductors in air and review the various approaches for improving their stability in air. The first part is focusing on 2D metals. The confined growth of 2D metals at graphene/SiC interface paves a way to synthesize air-stable 2D metals with unique optical and electronic properties. Due to the antioxidation of 2D metals under graphene, more ex situ characterization methods can be applied to explore more exciting properties. CHet process enables synthesis of millimeter-scale 2D metals,^77,79,117^ including Ga, In, Ag, and Pb, which is vital to further device fabrication. Furthermore, the capping graphene layer is well-known as a good platform to integrate other materials to form novel heterostructures.^118,119^ Integrating these air-stable 2D metals/EG systems with other materials may also lead to unexpected phenomena and performance. There are already some pioneering works in this field.^116,120^ For example, Li et al.^116^ grew (Bi,Sb)_2_Te_3_ on top of graphene/Ga by molecular beam epitaxy to create an epitaxial (Bi,Sb)_2_Te_3_/graphene/Ga heterostructure (Figure 5e) and verified the atomically sharp interfaces between each material with TEM (Figure 5f). Due to the protection of graphene, 2D Ga was not degraded after the growth of (Bi,Sb)_2_Te_3_. The proximity-induced superconductivity of (Bi,Sb)_2_Te_3_/graphene/Ga heterostructure was investigated with graphite and hBN-integrated tunnel junction devices and transport tunneling spectroscopy measurements.^116^ They found a robust proximity-induced superconducting gap in the Dirac surface states of topological insulating (Bi,Sb)_2_Te_3_ films and the presence of Abrikosov vortices in tunneling conductance down to a single vortex in the heterostructure.^116^ Additionally, there are still a lot of 2D metals, which have been predicted to have intriguing properties, not experimentally synthesized. For example, DFT results show that some nonmagnetic 3D elemental metals (such as Ru, Pd and Ti) will become magnetic in their 2D counterparts due to coordination number decreases and energy band narrowing of the out-of-plane orbitals.^121^ These confined 2D monoelemental metals can also be a platform to prepare new confined 2D alloys, oxide and nitride. For instance, Rajabpour et al.^122^ synthesized 2D In_*x*_Ga_1–*x*_ (0 < x < 1) alloys with controllable composition via changing the elemental concentration in the precursor during CHet. The 2D In_*x*_Ga_1–*x*_ alloys are verified to be continuous across microsize terraces of SiC and uniformly distributed in the interface without segregation. Notably, the dielectric function and superconductivity of 2D In_*x*_Ga_1–*x*_ alloys can be tuned by alloy compositions.

And for 2D semiconductors, group-VI 2D TMDCs have emerged as relatively air-stable 2D semiconductors and have been extensively studied. The presence of grain boundaries and point defects makes these materials sensitive to air, leading to rapid oxidation and thus decreasing stability. To address these issues, several strategies have been proposed. Long-range-order vdW epitaxy effectively eliminates high-angle boundaries, the unidirectionally oriented TMDCs obtained by engineered substrates reduced most of the antiphase boundaries.^123−127^ Utilizing postgrowth annealing under a chalcogen-rich environment, or substitutional doping in the lattice drastically reduced chalcogen vacancies.^107^ Although these approaches are well-studied, the quality of CVD-TMDCs at the wafer scale still falls short of exfoliated materials. Hence, it is essential to study the impact of different CVD precursor species, particularly the MOCVD precursors which potentially can provide a high-quality thin film at larger scales. Meanwhile, several air-stable candidates such as PtSe_2_^128,129^ and PdSe_2_^130,131^ have been demonstrated; however, the development of large-scale growth methods and quality improvement are still in early phases. Conceptionally, adding a capping layer to encapsulate the air-sensitive materials is another avenue to improve their air stability, which may enable further applications on those high-performance but air-sensitive materials. Hence, to realize the electronic-grade, large-area, and air-stable semiconductors, it is crucial to not only develop growth methods for the materials, bu also t further investigate the sequential growth of scaled-up capping layers.

## References

[ref1] Chaves A.; Azadani J. G.; Alsalman H.; da Costa D. R.; Frisenda R.; Chaves A. J.; Song S. H.; Kim Y. D.; He D.; Zhou J.; Castellanos-Gomez A.; Peeters F. M.; Liu Z.; Hinkle C. L.; Oh S. H.; Ye P. D.; Koester S. J.; Lee Y. H.; Avouris P.; Wang X.; Low T. Bandgap Engineering of Two-Dimensional Semiconductor Materials. NPJ. 2D Mater. Appl. 2020, 4 (1), 29 10.1038/s41699-020-00162-4.

[ref2] Lei Y.; Zhang T.; Lin Y.-C.; Granzier-Nakajima T.; Bepete G.; Kowalczyk D. A.; Lin Z.; Zhou D.; Schranghamer T. F.; Dodda A.; Sebastian A.; Chen Y.; Liu Y.; Pourtois G.; Kempa T. J.; Schuler B.; Edmonds M. T.; Quek S. Y.; Wurstbauer U.; Wu S. M.; Glavin N. R.; Das S.; Dash S. P.; Redwing J. M.; Robinson J. A.; Terrones M. Graphene and Beyond: Recent Advances in Two-Dimensional Materials Synthesis, Properties, and Devices. ACS Nanoscience Au 2022, 2 (6), 450–485. 10.1021/acsnanoscienceau.2c00017. 36573124 PMC9782807

[ref3] Manzeli S.; Ovchinnikov D.; Pasquier D.; Yazyev O. v.; Kis A. 2D Transition Metal Dichalcogenides. Nat. Rev. Mater. 2017, 2, 17033 10.1038/natrevmats.2017.33.

[ref4] Carvalho A.; Wang M.; Zhu X.; Rodin A. S.; Su H.; Castro Neto A. H. Phosphorene: From Theory to Applications. Nat. Rev. Mater. 2016, 1 (11), 1–16. 10.1038/natrevmats.2016.61.

[ref5] Nan H. Y.; Ni Z. H.; Wang J.; Zafar Z.; Shi Z. X.; Wang Y. Y. The Thermal Stability of Graphene in Air Investigated by Raman Spectroscopy. J. Raman Spectrosc. 2013, 44 (7), 1018–1021. 10.1002/jrs.4312.

[ref6] Liu Z.; Gong Y.; Zhou W.; Ma L.; Yu J.; Idrobo J. C.; Jung J.; Macdonald A. H.; Vajtai R.; Lou J.; Ajayan P. M. Ultrathin High-Temperature Oxidation-Resistant Coatings of Hexagonal Boron Nitride. Nat. Commun. 2013, 4, 2541 10.1038/ncomms3541. 24092019

[ref7] Bhimanapati G. R.; Lin Z.; Meunier V.; Jung Y.; Cha J. J.; Das S.; Xiao D.; Son Y.; Strano M. S.; Cooper V. R.; Liang L.; Louie S. G.; Ringe E.; Zhou W.; Sumpter B. G.; Terrones H.; Xia F.; Wang Y.; Zhu J.; Akinwande D.; Alem N.; Schuller J. A.; Schaak R. E.; Terrones M.; Robinson J. A. Recent Advances in Two-Dimensional Materials Beyond Graphene. ACS Nano 2015, 9 (12), 11509–11539. 10.1021/acsnano.5b05556. 26544756

[ref8] Radisavljevic B.; Radenovic A.; Brivio J.; Giacometti V.; Kis A. Single-Layer MoS_2_ Transistors. Nat. Nanotechnol 2011, 6 (3), 147–150. 10.1038/nnano.2010.279. 21278752

[ref9] Ren Y.; Hu L.; Shao Y.; Hu Y.; Huang L.; Shi X. Magnetism of Elemental Two-Dimensional Metals. J. Mater. Chem. C Mater. 2021, 9 (13), 4554–4561. 10.1039/D1TC00438G.

[ref10] Zhang T.; Cheng P.; Li W. J.; Sun Y. J.; Wang G.; Zhu X. G.; He K.; Wang L.; Ma X.; Chen X.; Wang Y.; Liu Y.; Lin H. Q.; Jia J. F.; Xue Q. K. Superconductivity in One-Atomic-Layer Metal Films Grown on Si(111). Nat. Phys. 2010, 6 (2), 104–108. 10.1038/nphys1499.

[ref11] Wang T.; Park M.; Yu Q.; Zhang J.; Yang Y. Stability and Synthesis of 2D Metals and Alloys: A Review. Mater. Today Adv. 2020, 8, 100092 10.1016/j.mtadv.2020.100092.

[ref12] Yu Q.; Yang Y. Synthesis of Two-Dimensional Metallic Nanosheets: From Elemental Metals to Chemically Complex Alloys. ChemNanoMat 2020, 6 (12), 1683–1711. 10.1002/cnma.202000448.

[ref13] Ma Y.; Li B.; Yang S. Ultrathin Two-Dimensional Metallic Nanomaterials. Mater. Chem. Front 2018, 2 (3), 456–457. 10.1039/C7QM00548B.

[ref14] Ling T.; Wang J. J.; Zhang H.; Song S. T.; Zhou Y. Z.; Zhao J.; Du X. W. Freestanding Ultrathin Metallic Nanosheets: Materials, Synthesis, and Applications. Adv. Mater. 2015, 27 (36), 5396–5402. 10.1002/adma.201501403. 26214606

[ref15] Politano A.; Vitiello M. S.; Viti L.; Boukhvalov D. W.; Chiarello G. The Role of Surface Chemical Reactivity in the Stability of Electronic Nanodevices Based on Two-Dimensional Materials “beyond Graphene” and Topological Insulators. FlatChem. 2017, 1, 60–64. 10.1016/j.flatc.2016.11.003.

[ref16] Rasmussen F. A.; Thygesen K. S. Computational 2D Materials Database: Electronic Structure of Transition-Metal Dichalcogenides and Oxides. J. Phys. Chem. C 2015, 119 (23), 13169–13183. 10.1021/acs.jpcc.5b02950.

[ref17] Pető J.; Ollár T.; Vancsó P.; Popov Z. I.; Magda G. Z.; Dobrik G.; Hwang C.; Sorokin P. B.; Tapasztó L. Spontaneous Doping of the Basal Plane of MoS_2_ Single Layers through Oxygen Substitution under Ambient Conditions. Nat. Chem. 2018, 10 (12), 1246–1251. 10.1038/s41557-018-0136-2. 30224684

[ref18] Zhao J.; Zhou G.; Yan K.; Xie J.; Li Y.; Liao L.; Jin Y.; Liu K.; Hsu P. C.; Wang J.; Cheng H. M.; Cui Y. Air-Stable and Freestanding Lithium Alloy/Graphene Foil as an Alternative to Lithium Metal Anodes. Nat. Nanotechnol 2017, 12 (10), 993–999. 10.1038/nnano.2017.129. 28692059

[ref19] Preston D. J.; Mafra D. L.; Miljkovic N.; Kong J.; Wang E. N. Scalable Graphene Coatings for Enhanced Condensation Heat Transfer. Nano Lett. 2015, 15 (5), 2902–2909. 10.1021/nl504628s. 25826223

[ref20] Briggs N.; Gebeyehu Z. M.; Vera A.; Zhao T.; Wang K.; Duran A. D. L. F.; Bersch B.; Bowen T.; Knappenberger K. L.; Robinson J. A. Epitaxial Graphene/Silicon Carbide Intercalation: A Minireview on Graphene Modulation and Unique 2D Materials. Nanoscale 2019, 11 (33), 15440–15447. 10.1039/C9NR03721G. 31393495

[ref21] Briggs N.; Bersch B.; Wang Y.; Jiang J.; Koch R. J.; Nayir N.; Wang K.; Kolmer M.; Ko W.; de La Fuente Duran A.; Subramanian S.; Dong C.; Shallenberger J.; Fu M.; Zou Q.; Chuang Y. W.; Gai Z.; Li A. P.; Bostwick A.; Jozwiak C.; Chang C. Z.; Rotenberg E.; Zhu J.; van Duin A. C. T.; Crespi V.; Robinson J. A. Atomically Thin Half-van Der Waals Metals Enabled by Confinement Heteroepitaxy. Nat. Mater. 2020, 19, 637–643. 10.1038/s41563-020-0631-x. 32157191

[ref22] Liz-Marzán L. M.; Grzelczak M. Growing Anisotropic Crystals at the Nanoscale. Science 2017, 356 (6343), 1120–1121. 10.1126/science.aam8774. 28619898

[ref23] Wang F.; Wang Z.; Shifa T. A.; Wen Y.; Wang F.; Zhan X.; Wang Q.; Xu K.; Huang Y.; Yin L.; et al. Two-dimensional Non-layered Materials: Synthesis, Properties and Applications. Adv. Funct Mater. 2017, 27 (19), 1603254 10.1002/adfm.201603254.

[ref24] Kang Y.; Xue Q.; Jin P.; Jiang J.; Zeng J.; Chen Y. Rhodium Nanosheets-Reduced Graphene Oxide Hybrids: A Highly Active Platinum-Alternative Electrocatalyst for the Methanol Oxidation Reaction in Alkaline Media. ACS Sustain Chem. Eng. 2017, 5 (11), 10156–10162. 10.1021/acssuschemeng.7b02163.

[ref25] Gao S.; Lin Y.; Jiao X.; Sun Y.; Luo Q.; Zhang W.; Li D.; Yang J.; Xie Y. Partially Oxidized Atomic Cobalt Layers for Carbon Dioxide Electroreduction to Liquid Fuel. Nature 2016, 529 (7584), 68–71. 10.1038/nature16455. 26738592

[ref26] Kong X.; Xu K.; Zhang C.; Dai J.; Norooz Oliaee S.; Li L.; Zeng X.; Wu C.; Peng Z. Free-Standing Two-Dimensional Ru Nanosheets with High Activity toward Water Splitting. ACS Catal. 2016, 6 (3), 1487–1492. 10.1021/acscatal.5b02730.

[ref27] Bai J.; Xu G.-R.; Xing S.-H.; Zeng J.-H.; Jiang J.-X.; Chen Y. Hydrothermal Synthesis and Catalytic Application of Ultrathin Rhodium Nanosheet Nanoassemblies. ACS Appl. Mater. Interfaces 2016, 8 (49), 33635–33641. 10.1021/acsami.6b11210. 27960374

[ref28] Duan H.; Yan N.; Yu R.; Chang C.-R.; Zhou G.; Hu H.-S.; Rong H.; Niu Z.; Mao J.; Asakura H.; Tanaka T.; Dyson P. J.; Li J.; Li Y. Ultrathin Rhodium Nanosheets. Nat. Commun. 2014, 5 (1), 3093 10.1038/ncomms4093. 24435210

[ref29] Xu D.; Liu X.; Lv H.; Liu Y.; Zhao S.; Han M.; Bao J.; He J.; Liu B. Ultrathin Palladium Nanosheets with Selectively Controlled Surface Facets. Chem. Sci. 2018, 9 (19), 4451–4455. 10.1039/C8SC00605A. 29896386 PMC5956979

[ref30] Zhang Q.; Hu Y.; Guo S.; Goebl J.; Yin Y. Seeded Growth of Uniform Ag Nanoplates with High Aspect Ratio and Widely Tunable Surface Plasmon Bands. Nano Lett. 2010, 10 (12), 5037–5042. 10.1021/nl1032233. 21038884

[ref31] Kim B.-H.; Oh J.-H.; Han S. H.; Yun Y.-J.; Lee J.-S. Combinatorial Polymer Library Approach for the Synthesis of Silver Nanoplates. Chem. Mater. 2012, 24 (22), 4424–4433. 10.1021/cm3028115.

[ref32] Xu D.; Liu Y.; Zhao S.; Lu Y.; Han M.; Bao J. Novel Surfactant-Directed Synthesis of Ultra-Thin Palladium Nanosheets as Efficient Electrocatalysts for Glycerol Oxidation. Chem. Commun. 2017, 53 (10), 1642–1645. 10.1039/C6CC08953D. 28098268

[ref33] Jang K.; Kim H. J.; Son S. U. Low-Temperature Synthesis of Ultrathin Rhodium Nanoplates via Molecular Orbital Symmetry Interaction between Rhodium Precursors. Chem. Mater. 2010, 22 (4), 1273–1275. 10.1021/cm902948v.

[ref34] Jang M. H.; Kim J. K.; Tak H.; Yoo H. Controllable Synthesis of Multi-Layered Gold Spirangles. J. Mater. Chem. 2011, 21 (44), 17606–17608. 10.1039/c1jm13531g.

[ref35] Goebl J.; Zhang Q.; He L.; Yin Y. Monitoring the Shape Evolution of Silver Nanoplates: A Marker Study. Angewandte Chemie - International Edition 2012, 51 (2), 552–555. 10.1002/anie.201107240. 22125270

[ref36] Shahjamali M. M.; Bosman M.; Cao S.; Huang X.; Saadat S.; Martinsson E.; Aili D.; Tay Y. Y.; Liedberg B.; Loo S. C. J.; Zhang H.; Boey F.; Xue C. Gold Coating of Silver Nanoprisms. Adv. Funct Mater. 2012, 22 (4), 849–854. 10.1002/adfm.201102028.

[ref37] Ge J.; He D.; Chen W.; Ju H.; Zhang H.; Chao T.; Wang X.; You R.; Lin Y.; Wang Y.; Zhu J.; Li H.; Xiao B.; Huang W.; Wu Y.; Hong X.; Li Y. Atomically Dispersed Ru on Ultrathin Pd Nanoribbons. J. Am. Chem. Soc. 2016, 138 (42), 13850–13853. 10.1021/jacs.6b09246. 27740759

[ref38] Zeng J.; Xia X.; Rycenga M.; Henneghan P.; Li Q.; Xia Y. Successive Deposition of Silver on Silver Nanoplates: Lateral versus Vertical Growth. Angewandte Chemie - International Edition 2011, 50 (1), 244–249. 10.1002/anie.201005549. 21038402

[ref39] Li Z.; Lv W.; Zhang C.; Qin J.; Wei W.; Shao J.-J.; Wang D.-W.; Li B.; Kang F.; Yang Q.-H. Nanospace-Confined Formation of Flattened Sn Sheets in Pre-Seeded Graphenes for Lithium Ion Batteries. Nanoscale 2014, 6 (16), 9554–9558. 10.1039/C4NR01924E. 24993388

[ref40] Wang L.; Zhu Y.; Wang J.-Q.; Liu F.; Huang J.; Meng X.; Basset J.-M.; Han Y.; Xiao F.-S. Two-Dimensional Gold Nanostructures with High Activity for Selective Oxidation of Carbon-Hydrogen Bonds. Nat. Commun. 2015, 6 (1), 6957 10.1038/ncomms7957. 25902034 PMC4421807

[ref41] Jiang Y.; Yan Y.; Chen W.; Khan Y.; Wu J.; Zhang H.; Yang D. Single-Crystalline Pd Square Nanoplates Enclosed by (100) Facets on Reduced Graphene Oxide for Formic Acid Electro-Oxidation. Chem. Commun. 2016, 52 (99), 14204–14207. 10.1039/C6CC08464H. 27840881

[ref42] Huang X.; Li S.; Huang Y.; Wu S.; Zhou X.; Li S.; Gan C. L.; Boey F.; Mirkin C. A.; Zhang H. Synthesis of Hexagonal Close-Packed Gold Nanostructures. Nat. Commun. 2011, 2 (1), 292 10.1038/ncomms1291. 21522136

[ref43] Yang S.; Qiu P.; Yang G. Graphene Induced Formation of Single Crystal Pt Nanosheets through 2-Dimensional Aggregation and Sintering of Nanoparticles in Molten Salt Medium. Carbon 2014, 77, 1123–1131. 10.1016/j.carbon.2014.06.030.

[ref44] Zhao J.; Deng Q.; Bachmatiuk A.; Sandeep G.; Popov A.; Eckert J.; Rümmeli M. H. Free-Standing Single-Atom-Thick Iron Membranes Suspended in Graphene Pores. Science 2014, 343 (6176), 1228–1232. 10.1126/science.1245273. 24626924

[ref45] Qin H. L.; Wang D.; Huang Z. L.; Wu D. M.; Zeng Z. C.; Ren B.; Xu K.; Jin J. Thickness-Controlled Synthesis of Ultrathin Au Sheets and Surface Plasmonic Property. J. Am. Chem. Soc. 2013, 135 (34), 12544–12547. 10.1021/ja406107u. 23941113

[ref46] Fan Z.; Huang X.; Han Y.; Bosman M.; Wang Q.; Zhu Y.; Liu Q.; Li B.; Zeng Z.; Wu J.; Shi W.; Li S.; Gan C. L.; Zhang H. Surface Modification-Induced Phase Transformation of Hexagonal Close-Packed Gold Square Sheets. Nat. Commun. 2015, 6 (1), 6571 10.1038/ncomms7571. 25766635

[ref47] Hong X.; Tan C.; Liu J.; Yang J.; Wu X.-J.; Fan Z.; Luo Z.; Chen J.; Zhang X.; Chen B.; Zhang H. AuAg Nanosheets Assembled from Ultrathin AuAg Nanowires. J. Am. Chem. Soc. 2015, 137 (4), 1444–1447. 10.1021/ja513120u. 25597345

[ref48] Derivaz M.; Dentel D.; Stephan R.; Hanf M.-C.; Mehdaoui A.; Sonnet P.; Pirri C. Continuous Germanene Layer on Al(111). Nano Lett. 2015, 15 (4), 2510–2516. 10.1021/acs.nanolett.5b00085. 25802988

[ref49] Wu X.; Shao Y.; Liu H.; Feng Z.; Wang Y.-L.; Sun J.-T.; Liu C.; Wang J.-O.; Liu Z.-L.; Zhu S.-Y.; Wang Y.-Q.; Du S.-X.; Shi Y.-G.; Ibrahim K.; Gao H.-J. Epitaxial Growth and Air-Stability of Monolayer Antimonene on PdTe_2_. Adv. Mater. 2017, 29 (11), 1605407 10.1002/adma.201605407. 28028843

[ref50] Yuhara J.; Fujii Y.; Nishino K.; Isobe N.; Nakatake M.; Xian L.; Rubio A.; Le Lay G. Large Area Planar Stanene Epitaxially Grown on Ag(111). 2d Mater. 2018, 5 (2), 025002 10.1088/2053-1583/aa9ea0.

[ref51] Zhuang J.; Gao N.; Li Z.; Xu X.; Wang J.; Zhao J.; Dou S. X.; Du Y. Cooperative Electron-Phonon Coupling and Buckled Structure in Germanene on Au(111). ACS Nano 2017, 11 (4), 3553–3559. 10.1021/acsnano.7b00687. 28221757

[ref52] Zhu F.; Chen W.; Xu Y.; Gao C.; Guan D.; Liu C.; Qian D.; Zhang S.-C.; Jia J. Epitaxial Growth of Two-Dimensional Stanene. Nat. Mater. 2015, 14 (10), 1020–1025. 10.1038/nmat4384. 26237127

[ref53] Reis F.; Li G.; Dudy L.; Bauernfeind M.; Glass S.; Hanke W.; Thomale R.; Schäfer J.; Claessen R. Bismuthene on a SiC Substrate: A Candidate for a High-Temperature Quantum Spin Hall Material. Science (1979) 2017, 357 (6348), 287–290. 10.1126/science.aai8142. 28663438

[ref54] Hussain N.; Liang T.; Zhang Q.; Anwar T.; Huang Y.; Lang J.; Huang K.; Wu H. Ultrathin Bi Nanosheets with Superior Photoluminescence. Small 2017, 13 (36), 1701349 10.1002/smll.201701349. 28762634

[ref55] Gu J.; Li B.; Du Z.; Zhang C.; Zhang D.; Yang S. Multi-Atomic Layers of Metallic Aluminum for Ultralong Life Lithium Storage with High Volumetric Capacity. Adv. Funct Mater. 2017, 27 (27), 1700840 10.1002/adfm.201700840.

[ref56] Liu H.; Tang H.; Fang M.; Si W.; Zhang Q.; Huang Z.; Gu L.; Pan W.; Yao J.; Nan C.; et al. 2D Metals by Repeated Size Reduction. Adv. Mater. 2016, 28 (37), 8170–8176. 10.1002/adma.201601180. 27417413

[ref57] Wang T.; He Q.; Zhang J.; Ding Z.; Li F.; Yang Y. The Controlled Large-Area Synthesis of Two Dimensional Metals. Mater. Today 2020, 36, 30–39. 10.1016/j.mattod.2020.02.003.

[ref58] Funatsu A.; Tateishi H.; Hatakeyama K.; Fukunaga Y.; Taniguchi T.; Koinuma M.; Matsuura H.; Matsumoto Y. Synthesis of Monolayer Platinum Nanosheets. Chem. Commun. 2014, 50 (62), 8503–8506. 10.1039/C4CC02527J. 24947470

[ref59] Gu J.; Du Z.; Zhang C.; Ma J.; Li B.; Yang S. Liquid-Phase Exfoliated Metallic Antimony Nanosheets toward High Volumetric Sodium Storage. Adv. Energy Mater. 2017, 7 (17), 1700447 10.1002/aenm.201700447.

[ref60] Fukuda K.; Sato J.; Saida T.; Sugimoto W.; Ebina Y.; Shibata T.; Osada M.; Sasaki T. Fabrication of Ruthenium Metal Nanosheets via Topotactic Metallization of Exfoliated Ruthenate Nanosheets. Inorg. Chem. 2013, 52 (5), 2280–2282. 10.1021/ic302720d. 23421819

[ref61] Chen Y.; Fan Z.; Zhang Z.; Niu W.; Li C.; Yang N.; Chen B.; Zhang H. Two-Dimensional Metal Nanomaterials: Synthesis, Properties, and Applications. Chem. Rev. 2018, 118 (13), 6409–6455. 10.1021/acs.chemrev.7b00727. 29927583

[ref62] Nilius N. Properties of Oxide Thin Films and Their Adsorption Behavior Studied by Scanning Tunneling Microscopy and Conductance Spectroscopy. Surf. Sci. Rep 2009, 64 (12), 595–659. 10.1016/j.surfrep.2009.07.004.

[ref63] Ding R.; Li W.; Wang X.; Gui T.; Li B.; Han P.; Tian H.; Liu A.; Wang X.; Liu X.; Gao X.; Wang W.; Song L. A Brief Review of Corrosion Protective Films and Coatings Based on Graphene and Graphene Oxide. J. Alloys Compd. 2018, 764, 1039–1055. 10.1016/j.jallcom.2018.06.133.

[ref64] Kavan L.; Yum J. H.; Grätzel M. Optically Transparent Cathode for Dye-Sensitized Solar Cells Based on Graphene Nanoplatelets. ACS Nano 2011, 5 (1), 165–172. 10.1021/nn102353h. 21126092

[ref65] Wassei J. K.; Kaner R. B. Graphene, a Promising Transparent Conductor. Mater. Today 2010, 13 (3), 52–59. 10.1016/S1369-7021(10)70034-1.

[ref66] Qi Y.; Wang Y.; Pang Z.; Dou Z.; Wei T.; Gao P.; Zhang S.; Xu X.; Chang Z.; Deng B.; Chen S.; Chen Z.; Ci H.; Wang R.; Zhao F.; Yan J.; Yi X.; Liu K.; Peng H.; Liu Z.; Tong L.; Zhang J.; Wei Y.; Li J.; Liu Z. Fast Growth of Strain-Free AlN on Graphene-Buffered Sapphire. J. Am. Chem. Soc. 2018, 140 (38), 11935–11941. 10.1021/jacs.8b03871. 30175921

[ref67] Kim Y.; Cruz S. S.; Lee K.; Alawode B. O.; Choi C.; Song Y.; Johnson J. M.; Heidelberger C.; Kong W.; Choi S.; Qiao K.; Almansouri I.; Fitzgerald E. A.; Kong J.; Kolpak A. M.; Hwang J.; Kim J. Remote Epitaxy through Graphene Enables Two-Dimensional Material-Based Layer Transfer. Nature 2017, 544 (7650), 340–343. 10.1038/nature22053. 28426001

[ref68] Jiang J.; Sun X.; Chen X.; Wang B.; Chen Z.; Hu Y.; Guo Y.; Zhang L.; Ma Y.; Gao L.; Zheng F.; Jin L.; Chen M.; Ma Z.; Zhou Y.; Padture N. P.; Beach K.; Terrones H.; Shi Y.; Gall D.; Lu T. M.; Wertz E.; Feng J.; Shi J. Carrier Lifetime Enhancement in Halide Perovskite via Remote Epitaxy. Nat. Commun. 2019, 10 (1), 4145 10.1038/s41467-019-12056-1. 31515482 PMC6742762

[ref69] Jeong J.; Min K. A.; Kang B. K.; Shin D. H.; Yoo J.; Yang W. S.; Lee S. W.; Hong S.; Hong Y. J. Remote Heteroepitaxy across Graphene: Hydrothermal Growth of Vertical ZnO Microrods on Graphene-Coated GaN Substrate. Appl. Phys. Lett. 2018, 113 (23), 233103 10.1063/1.5064542.

[ref70] Kim H.; Chang C. S.; Lee S.; Jiang J.; Jeong J.; Park M.; Meng Y.; Ji J.; Kwon Y.; Sun X.; Kong W.; Kum H. S.; Bae S.-H.; Lee K.; Hong Y. J.; Shi J.; Kim J. Remote Epitaxy. Nature Reviews Methods Primers 2022, 2 (1), 40 10.1038/s43586-022-00122-w.

[ref71] Riedl C.; Coletti C.; Starke U. Structural and Electronic Properties of Epitaxial Graphene on SiC(0001): A Review of Growth, Characterization, Transfer Doping and Hydrogen Intercalation. J. Phys. D Appl. Phys. 2010, 43, 374009 10.1088/0022-3727/43/37/374009.

[ref72] Robinson J.; Weng X.; Trumbull K.; Cavalero R.; Wetherington M.; Frantz E.; LaBella M.; Hughes Z.; Fanton M.; Snyder D. Nucleation of Epitaxial Graphene on SiC(0001). ACS Nano 2010, 4 (1), 153–158. 10.1021/nn901248j. 20000439

[ref73] Geim A. K. Graphene: Status and Prospects. Science 2009, 324, 1530–1534. 10.1126/science.1158877. 19541989

[ref74] Robinson J. A.; Hollander M.; LaBella M.; Trumbull K. A.; Cavalero R.; Snyder D. W. Epitaxial Graphene Transistors: Enhancing Performance via Hydrogen Intercalation. Nano Lett. 2011, 11 (9), 3875–3880. 10.1021/nl2019855. 21805993

[ref75] Riedl C.; Coletti C.; Iwasaki T.; Zakharov A. A.; Starke U. Quasi-Free-Standing Epitaxial Graphene on SiC Obtained by Hydrogen Intercalation. Phys. Rev. Lett. 2009, 103, 246804 10.1103/PhysRevLett.103.246804. 20366220

[ref76] Rosenzweig P.; Karakachian H.; Link S.; Küster K.; Starke U. Tuning the Doping Level of Graphene in the Vicinity of the Van Hove Singularity via Ytterbium Intercalation. Phys. Rev. B 2019, 100 (3), 35445 10.1103/PhysRevB.100.035445.

[ref77] Briggs N.; Bersch B.; Wang Y.; Jiang J.; Koch R. J.; Nayir N.; Wang K.; Kolmer M.; Ko W.; De La Fuente Duran A.; Subramanian S.; Dong C.; Shallenberger J.; Fu M.; Zou Q.; Chuang Y. W.; Gai Z.; Li A. P.; Bostwick A.; Jozwiak C.; Chang C. Z.; Rotenberg E.; Zhu J.; van Duin A. C. T.; Crespi V.; Robinson J. A. Atomically Thin Half-van Der Waals Metals Enabled by Confinement Heteroepitaxy. Nat. Mater. 2020, 19 (6), 637–643. 10.1038/s41563-020-0631-x. 32157191

[ref78] Wang Y.; Crespi V. H. Atlas of 2D Metals Epitaxial to SiC: Filling-Controlled Gapping Conditions and Alloying Rules. arXiv (Materials Science), November 3, 2020, ver 1. 10.48550/arXiv.2011.01914.

[ref79] Lee W.; Wang Y.; Qin W.; Kim H.; Liu M.; Nunley T. N.; Fang B.; Maniyara R.; Dong C.; Robinson J. A.; Crespi V. H.; Li X.; MacDonald A. H.; Shih C.-K. Confined Monolayer Ag As a Large Gap 2D Semiconductor and Its Momentum Resolved Excited States. Nano Lett. 2022, 22 (19), 7841 10.1021/acs.nanolett.2c02501. 36126277

[ref80] Forti S.; Link S.; Stöhr A.; Niu Y.; Zakharov A. A.; Coletti C.; Starke U. Semiconductor to Metal Transition in Two-Dimensional Gold and Its van Der Waals Heterostack with Graphene. Nat. Commun. 2020, 11, 2236 10.1038/s41467-020-15683-1. 32376867 PMC7203110

[ref81] Ichinokura S.; Sugawara K.; Takayama A.; Takahashi T.; Hasegawa S. Superconducting Calcium-Intercalated Bilayer Graphene. ACS Nano 2016, 10 (2), 2761–2765. 10.1021/acsnano.5b07848. 26815333

[ref82] Toyama H.; Akiyama R.; Ichinokura S.; Hashizume M.; Iimori T.; Endo Y.; Hobara R.; Matsui T.; Horii K.; Sato S.; Hirahara T.; Komori F.; Hasegawa S. Two-Dimensional Superconductivity of Ca-Intercalated Graphene on SiC: Vital Role of the Interface between Monolayer Graphene and the Substrate. ACS Nano 2022, 16 (3), 3582–3592. 10.1021/acsnano.1c11161. 35209713

[ref83] Li K.; Feng X.; Zhang W.; Ou Y.; Chen L.; He K.; Wang L. L.; Guo L.; Liu G.; Xue Q. K.; Ma X. Superconductivity in Ca-Intercalated Epitaxial Graphene on Silicon Carbide. Appl. Phys. Lett. 2013, 103 (6), 062601 10.1063/1.4817781.

[ref84] Endo Y.; Fukaya Y.; Mochizuki I.; Takayama A.; Hyodo T.; Hasegawa S. Structure of Superconducting Ca-Intercalated Bilayer Graphene/SiC Studied Using Total-Reflection High-Energy Positron Diffraction. Carbon 2020, 157, 857–862. 10.1016/j.carbon.2019.10.070.

[ref85] Wang X.; Liu N.; Wu Y.; Qu Y.; Zhang W.; Wang J.; Guan D.; Wang S.; Zheng H.; Li Y.; Liu C.; Jia J. Strong Coupling Superconductivity in Ca-Intercalated Bilayer Graphene on SiC. Nano Lett. 2022, 22 (18), 7651–7658. 10.1021/acs.nanolett.2c02804. 36066512

[ref86] Zhang X.; Tan Q.-H.; Wu J.-B.; Shi W.; Tan P.-H. Review on the Raman Spectroscopy of Different Types of Layered Materials. Nanoscale 2016, 8 (12), 6435–6450. 10.1039/C5NR07205K. 26955865

[ref87] Wetherington M. T.; Turker F.; Bowen T.; Vera A.; Rajabpour S.; Briggs N.; Subramanian S.; Maloney A.; Robinson J. A. 2-Dimensional Polar Metals: A Low-Frequency Raman Scattering Study. 2d Mater. 2021, 8 (4), 041003 10.1088/2053-1583/ac2245.

[ref88] He W.; Wetherington M. T.; Ulman K. A.; Gray J. L.; Robinson J. A.; Quek S. Y. Shear Modes in a 2D Polar Metal. J. Phys. Chem. Lett. 2022, 13 (18), 4015–4020. 10.1021/acs.jpclett.2c00719. 35485838

[ref89] He W.; Wetherington M. T.; Ulman K. A.; Robinson J. A.; Quek S. Y. Interface-Mediated Resonant Raman Enhancement for Shear Modes in a 2D Polar Metal. J. Phys. Chem. C 2022, 126 (34), 14581–14589. 10.1021/acs.jpcc.2c04433. 35485838

[ref90] Steves M. A.; Wang Y.; Briggs N.; Zhao T.; El-Sherif H.; Bersch B. M.; Subramanian S.; Dong C.; Bowen T.; Fuente Duran A. D. La; Nisi K.; Lassaunière M.; Wurstbauer U.; Bassim N. D.; Fonseca J.; Robinson J. T.; Crespi V. H.; Robinson J.; Knappenberger K. L. Unexpected Near-Infrared to Visible Nonlinear Optical Properties from 2-D Polar Metals. Nano Lett. 2020, 20 (11), 8312–8318. 10.1021/acs.nanolett.0c03481. 33079555

[ref91] Steves M. A.; Rajabpour S.; Wang K.; Dong C.; He W.; Quek S. Y.; Robinson J. A.; Knappenberger K. L. Atomic-Level Structure Determines Electron-Phonon Scattering Rates in 2-D Polar Metal Heterostructures. ACS Nano 2021, 15 (11), 17780–17789. 10.1021/acsnano.1c05944. 34665593

[ref92] Nisi K.; Subramanian S.; He W.; Ulman K. A.; El-Sherif H.; Sigger F.; Lassaunière M.; Wetherington M. T.; Briggs N.; Gray J.; Holleitner A. W.; Bassim N.; Quek S. Y.; Robinson J. A.; Wurstbauer U. Light-Matter Interaction in Quantum Confined 2D Polar Metals. Adv. Funct Mater. 2021, 31 (4), 2005977 10.1002/adfm.202005977.

[ref93] Liu Y.; Duan X.; Shin H.-J.; Park S.; Huang Y.; Duan X. Promises and Prospects of Two-Dimensional Transistors. Nature 2021, 591 (7848), 43–53. 10.1038/s41586-021-03339-z. 33658691

[ref94] Cai H.; Gu Y.; Lin Y. C.; Yu Y.; Geohegan D. B.; Xiao K. Synthesis and Emerging Properties of 2D Layered III-VI Metal Chalcogenides. Appl. Phys. Rev. 2019, 6 (4), 041312 10.1063/1.5123487.

[ref95] Longo R. C.; Addou R.; Santosh K. C.; Noh J. Y.; Smyth C. M.; Barrera D.; Zhang C.; Hsu J. W. P.; Wallace R. M.; Cho K. Intrinsic Air Stability Mechanisms of Two-Dimensional Transition Metal Dichalcogenide Surfaces: Basal versus Edge Oxidation. 2d Mater. 2017, 4 (2), 025050 10.1088/2053-1583/aa636c.

[ref96] Chae S. H.; Jin Y.; Kim T. S.; Chung D. S.; Na H.; Nam H.; Kim H.; Perello D. J.; Jeong H. Y.; Ly T. H.; Lee Y. H. Oxidation Effect in Octahedral Hafnium Disulfide Thin Film. ACS Nano 2016, 10 (1), 1309–1316. 10.1021/acsnano.5b06680. 26735305

[ref97] Mleczko M. J.; Zhang C.; Lee H. R.; Kuo H.-H.; Magyari-Köpe B.; Moore R. G.; Shen Z.-X.; Fisher I. R.; Nishi Y.; Pop E. HfSe_2_ and ZrSe_2_: Two-Dimensional Semiconductors with Native High-κ Oxides. Sci. Adv. 2017, 3, e1700481 10.1126/sciadv.1700481. 28819644 PMC5553816

[ref98] Jo S. S.; Singh A.; Yang L.; Tiwari S. C.; Hong S.; Krishnamoorthy A.; Sales M. G.; Oliver S. M.; Fox J.; Cavalero R. L.; et al. Growth Kinetics and Atomistic Mechanisms of Native Oxidation of ZrS_x_Se_2-x_ and MoS_2_ Crystals. Nano Lett. 2020, 20 (12), 8592–8599. 10.1021/acs.nanolett.0c03263. 33180506

[ref99] Pace S.; Martini L.; Convertino D.; Keum D. H.; Forti S.; Pezzini S.; Fabbri F.; Mišeikis V.; Coletti C. Synthesis of Large-Scale Monolayer 1T′-MoTe_2_ and Its Stabilization via Scalable HBN Encapsulation. ACS Nano 2021, 15 (3), 4213–4225. 10.1021/acsnano.0c05936. 33605730 PMC8023802

[ref100] Wang Q. H.; Kalantar-Zadeh K.; Kis A.; Coleman J. N.; Strano M. S. Electronics and Optoelectronics of Two-Dimensional Transition Metal Dichalcogenides. Nat. Nanotechnol 2012, 7 (11), 699–712. 10.1038/nnano.2012.193. 23132225

[ref101] Pető J.; Ollár T.; Vancsó P.; Popov Z. I.; Magda G. Z.; Dobrik G.; Hwang C.; Sorokin P. B.; Tapasztó L. Spontaneous Doping of the Basal Plane of MoS_2_ Single Layers through Oxygen Substitution under Ambient Conditions. Nat. Chem. 2018, 10 (12), 1246–1251. 10.1038/s41557-018-0136-2. 30224684

[ref102] Politano A.; Chiarello G.; Kuo C. N.; Lue C. S.; Edla R.; Torelli P.; Pellegrini V.; Boukhvalov D. W. Tailoring the Surface Chemical Reactivity of Transition-Metal Dichalcogenide PtTe_2_ Crystals. Adv. Funct Mater. 2018, 28 (15), 1706504 10.1002/adfm.201706504.

[ref103] Liang Q.; Wang Q.; Zhang Q.; Wei J.; Lim S. X.; Zhu R.; Hu J.; Wei W.; Lee C.; Sow C.; Zhang W.; Wee A. T. S. High-Performance, Room Temperature, Ultra-Broadband Photodetectors Based on Air-Stable PdSe_2_. Adv. Mater. 2019, 31 (24), 1807609 10.1002/adma.201807609. 31025440

[ref104] Wang G.; Wang Z.; McEvoy N.; Fan P.; Blau W. J. Layered PtSe_2_ for Sensing, Photonic, and (Opto-)Electronic Applications. Adv. Mater. 2021, 33 (1), 2004070 10.1002/adma.202004070. 33225525

[ref105] Kang K.; Xie S.; Huang L.; Han Y.; Huang P. Y.; Mak K. F.; Kim C.-J.; Muller D.; Park J. High-Mobility Three-Atom-Thick Semiconducting Films with Wafer-Scale Homogeneity. Nature 2015, 520 (7549), 656–660. 10.1038/nature14417. 25925478

[ref106] Eichfeld S. M.; Hossain L.; Lin Y.-C.; Piasecki A. F.; Kupp B.; Birdwell A. G.; Burke R. A.; Lu N.; Peng X.; Li J.; Azcatl A.; McDonnell S.; Wallace R. M.; Kim M. J.; Mayer T. S.; Redwing J. M.; Robinson J. A. Highly Scalable, Atomically Thin WSe_2_ Grown via Metal-Organic Chemical Vapor Deposition. ACS Nano 2015, 9 (2), 2080–2087. 10.1021/nn5073286. 25625184

[ref107] Lin Y.-C.; Jariwala B.; Bersch B. M.; Xu K.; Nie Y.; Wang B.; Eichfeld S. M.; Zhang X.; Choudhury T. H.; Pan Y.; Addou R.; Smyth C. M.; Li J.; Zhang K.; Haque M. A.; Fölsch S.; Feenstra R. M.; Wallace R. M.; Cho K.; Fullerton-Shirey S. K.; Redwing J. M.; Robinson J. A. Realizing Large-Scale, Electronic-Grade Two-Dimensional Semiconductors. ACS Nano 2018, 12 (2), 965–975. 10.1021/acsnano.7b07059. 29360349

[ref108] Wan Y.; Li E.; Yu Z.; Huang J. K.; Li M. Y.; Chou A. S.; Lee Y. te; Lee C. J.; Hsu H. C.; Zhan Q.; Aljarb A.; Fu J. H.; Chiu S. P.; Wang X.; Lin J. J.; Chiu Y. P.; Chang W. H.; Wang H.; Shi Y.; Lin N.; Cheng Y.; Tung V.; Li L. J. Low-Defect-Density WS_2_ by Hydroxide Vapor Phase Deposition. Nat. Commun. 2022, 13 (1), 4149 10.1038/s41467-022-31886-0. 35851038 PMC9293887

[ref109] Li X.; Puretzky A. A.; Sang X.; KC S.; Tian M.; Ceballos F.; Mahjouri-Samani M.; Wang K.; Unocic R. R.; Zhao H.; Duscher G.; Cooper V. R.; Rouleau C. M.; Geohegan D. B.; Xiao K. Suppression of Defects and Deep Levels Using Isoelectronic Tungsten Substitution in Monolayer MoSe_2_. Adv. Funct Mater. 2017, 27 (19), 1603850 10.1002/adfm.201603850.

[ref110] Torsi R.; Munson K. T.; Pendurthi R.; Marques E.; Van Troeye B.; Huberich L.; Schuler B.; Feidler M.; Wang K.; Pourtois G.; Das S.; Asbury J. B.; Lin Y.-C.; Robinson J. A. Dilute Rhenium Doping and Its Impact on Defects in MoS_2_. ACS Nano 2023, 17 (16), 15629–15640. 10.1021/acsnano.3c02626. 37534591

[ref111] Chuang S.; Battaglia C.; Azcatl A.; McDonnell S.; Kang J. S.; Yin X.; Tosun M.; Kapadia R.; Fang H.; Wallace R. M.; Javey A. MoS_2_ P-Type Transistors and Diodes Enabled by High Work Function MoO_x_ Contacts. Nano Lett. 2014, 14 (3), 1337–1342. 10.1021/nl4043505. 24568656

[ref112] Bi K.; Wan Q.; Shu Z.; Shao G.; Jin Y.; Zhu M.; Lin J.; Liu H.; Liu H.; Chen Y.; Liu S.; Duan H. High-Performance Lateral MoS_2_-MoO_3_ Heterojunction Phototransistor Enabled by in-Situ Chemical-Oxidation. Sci. China Mater. 2020, 63 (6), 1076–1084. 10.1007/s40843-019-1259-6.

[ref113] Shen P. C.; Lin Y.; Su C.; McGahan C.; Lu A. Y.; Ji X.; Wang X.; Wang H.; Mao N.; Guo Y.; Park J. H.; Wang Y.; Tisdale W.; Li J.; Ling X.; Aidala K. E.; Palacios T.; Kong J. Healing of Donor Defect States in Monolayer Molybdenum Disulfide Using Oxygen-Incorporated Chemical Vapour Deposition. Nat. Electron 2022, 5 (1), 28–36. 10.1038/s41928-021-00685-8.

[ref114] Kim J.-S.; Liu Y.; Zhu W.; Kim S.; Wu D.; Tao L.; Dodabalapur A.; Lai K.; Akinwande D. Toward Air-Stable Multilayer Phosphorene Thin-Films and Transistors. Sci. Rep 2015, 5 (1), 8989 10.1038/srep08989. 25758437 PMC4355728

[ref115] Zhao D.-H.; Gu Z.-H.; Wang T.-Y.; Guo X.-J.; Jiang X.-X.; Zhang M.; Zhu H.; Chen L.; Sun Q.-Q.; Zhang D. W. Sensitive MoS_2_ Photodetector Cell with High Air-Stability for Multifunctional in-Sensor Computing. Chip 2022, 1 (3), 100023 10.1016/j.chip.2022.100023.

[ref116] Li C.; Zhao Y.-F.; Vera A.; Lesser O.; Yi H.; Kumari S.; Yan Z.; Dong C.; Bowen T.; Wang K.; Wang H.; Thompson J. L.; Watanabe K.; Taniguchi T.; Reifsnyder Hickey D.; Oreg Y.; Robinson J. A.; Chang C.-Z.; Zhu J. Proximity-Induced Superconductivity in Epitaxial Topological Insulator/Graphene/Gallium Heterostructures. Nat. Mater. 2023, 22 (5), 570–575. 10.1038/s41563-023-01478-4. 36781950

[ref117] Vera A.; Yanez W.; Yang K.; Zheng B.; Dong C.; Wang Y.; Bowen T.; El-Sherif H.; Krishnan G.; Rajabpour S. Two-Dimensional Lead at the Graphene/Silicon Carbide Interface. arXiv (Materials Science), July 15, 2022, ver 2. 10.48550/arXiv.2205.06859.

[ref118] Solís-Fernández P.; Bissett M.; Ago H. Synthesis, Structure and Applications of Graphene-Based 2D Heterostructures. Chem. Soc. Rev. 2017, 46 (15), 4572–4613. 10.1039/C7CS00160F. 28691726

[ref119] Bhimanapati G. R.; Lin Z.; Meunier V.; Jung Y.; Cha J.; Das S.; Xiao D.; Son Y.; Strano M. S.; Cooper V. R.; Liang L.; Louie S. G.; Ringe E.; Zhou W.; Kim S. S.; Naik R. R.; Sumpter B. G.; Terrones H.; Xia F.; Wang Y.; Zhu J.; Akinwande D.; Alem N.; Schuller J. A.; Schaak R. E.; Terrones M.; Robinson J. A. Recent Advances in Two-Dimensional Materials beyond Graphene. ACS Nano 2015, 9 (12), 11509–11539. 10.1021/acsnano.5b05556. 26544756

[ref120] Rosenzweig P.; Karakachian H.; Marchenko D.; Küster K.; Starke U. Overdoping Graphene beyond the van Hove Singularity. Phys. Rev. Lett. 2020, 125 (17), 176403 10.1103/PhysRevLett.125.176403. 33156643

[ref121] Ren Y.; Hu L.; Shao Y.; Hu Y.; Huang L.; Shi X. Magnetism of Elemental Two-Dimensional Metals. J. Mater. Chem. C Mater. 2021, 9 (13), 4554–4561. 10.1039/D1TC00438G.

[ref122] Rajabpour S.; Vera A.; He W.; Katz B. N.; Koch R. J.; Lassaunière M.; Chen X.; Li C.; Nisi K.; El-Sherif H.; Wetherington M. T.; Dong C.; Bostwick A.; Jozwiak C.; van Duin A. C. T.; Bassim N.; Zhu J.; Wang G. C.; Wurstbauer U.; Rotenberg E.; Crespi V.; Quek S. Y.; Robinson J. A. Tunable 2D Group-III Metal Alloys. Adv. Mater. 2021, 33 (44), 2104265 10.1002/adma.202104265. 34480500

[ref123] Li T.; Guo W.; Ma L.; Li W.; Yu Z.; Han Z.; Gao S.; Liu L.; Fan D.; Wang Z.; et al. Epitaxial Growth of Wafer-Scale Molybdenum Disulfide Semiconductor Single Crystals on Sapphire. Nat. Nanotechnol 2021, 16 (11), 1201–1207. 10.1038/s41565-021-00963-8. 34475559

[ref124] Chubarov M.; Choudhury T. H.; Hickey D. R.; Bachu S.; Zhang T.; Sebastian A.; Bansal A.; Zhu H.; Trainor N.; Das S.; et al. Wafer-Scale Epitaxial Growth of Unidirectional WS_2_ Monolayers on Sapphire. ACS Nano 2021, 15 (2), 2532–2541. 10.1021/acsnano.0c06750. 33450158

[ref125] Zheng P.; Wei W.; Liang Z.; Qin B.; Tian J.; Wang J.; Qiao R.; Ren Y.; Chen J.; Huang C.; et al. Universal Epitaxy of Non-Centrosymmetric Two-Dimensional Single-Crystal Metal Dichalcogenides. Nat. Commun. 2023, 14 (1), 592 10.1038/s41467-023-36286-6. 36737606 PMC9898269

[ref126] Yang P.; Zhang S.; Pan S.; Tang B.; Liang Y.; Zhao X.; Zhang Z.; Shi J.; Huan Y.; Shi Y.; et al. Epitaxial Growth of Centimeter-Scale Single-Crystal MoS_2_ Monolayer on Au (111). ACS Nano 2020, 14 (4), 5036–5045. 10.1021/acsnano.0c01478. 32267670

[ref127] Wang J.; Xu X.; Cheng T.; Gu L.; Qiao R.; Liang Z.; Ding D.; Hong H.; Zheng P.; Zhang Z.; et al. Dual-Coupling-Guided Epitaxial Growth of Wafer-Scale Single-Crystal WS_2_ Monolayer on Vicinal a-Plane Sapphire. Nat. Nanotechnol 2022, 17 (1), 33–38. 10.1038/s41565-021-01004-0. 34782776

[ref128] Zhao Y.; Qiao J.; Yu Z.; Yu P.; Xu K.; Lau S. P.; Zhou W.; Liu Z.; Wang X.; Ji W.; Chai Y. High-Electron-Mobility and Air-Stable 2D Layered PtSe_2_ FETs. Adv. Mater. 2017, 29 (5), 1604230 10.1002/adma.201604230. 27886410

[ref129] Zhang Z. X.; Long-Hui Z.; Tong X. W.; Gao Y.; Xie C.; Tsang Y. H.; Luo L. B.; Wu Y. C. Ultrafast, Self-Driven, and Air-Stable Photodetectors Based on Multilayer PtSe_2_/Perovskite Heterojunctions. J. Phys. Chem. Lett. 2018, 9 (6), 1185–1194. 10.1021/acs.jpclett.8b00266. 29464954

[ref130] Oyedele A. D.; Yang S.; Liang L.; Puretzky A. A.; Wang K.; Zhang J.; Yu P.; Pudasaini P. R.; Ghosh A. W.; Liu Z.; Rouleau C. M.; Sumpter B. G.; Chisholm M. F.; Zhou W.; Rack P. D.; Geohegan D. B.; Xiao K. PdSe_2_: Pentagonal Two-Dimensional Layers with High Air Stability for Electronics. J. Am. Chem. Soc. 2017, 139 (40), 14090–14097. 10.1021/jacs.7b04865. 28873294

[ref131] Liang Q.; Wang Q.; Zhang Q.; Wei J.; Lim S. X.; Zhu R.; Hu J.; Wei W.; Lee C.; Sow C. H.; Zhang W.; Wee A. T. S. High-Performance, Room Temperature, Ultra-Broadband Photodetectors Based on Air-Stable PdSe_2_. Adv. Mater. 2019, 31 (24), 1807609 10.1002/adma.201807609. 31025440

